# Supplementary material for: Synthetic periphyton as a model system to understand species dynamics in complex microbial freshwater communities
Source: NPJ Biofilms Microbiomes. 2022 Jul 22;8:61. doi: 10.1038/s41522-022-00322-y (PMC9307524; doi:10.1038/s41522-022-00322-y)
Supplement: Supplementary file 1 — Supplementary Material [file 41522_2022_322_MOESM1_ESM.pdf]

Supplementary Method 1. Experimental design and measurements

The total number of chambers were inoculated at the start of each experiment and then used for the specific measurements. At sampling time point the same set of replicates was used for all the measurements, except for the confocal laser scanning microscopy. Thus, for the establishment of the synthetic periphyton at least 20 chambers ((5 replicates x 3 time points) + (5 replicates for CLSM)) and for the case study at least 120 chambers ((8 conditions x 4 replicates x 3 time points) + (8 conditions x 3 replicates for CLSM)) were inoculated. All measurements except the confocal microscopy were performed at three sampling times: at t_1 (4 days), t_2 (18 days) and t_3 (30 days). First, undisruptive measurements were performed (Supplementary Fig. 1a), followed by the disruptive measurements (Supplementary Fig. 1b), where supernatant and surface-attached cell fractions were analyzed:

- Physical structure measurements using OCT and photosystem II quantum yield with iPAM were performed on intact periphyton;
- Benthic biomass measurements with optical density and cell counter CASY, surface colonization area and microbial composition analysis with next-generation sequencing were performed on surface-attached biofilm after removal of the supernatant.

Confocal microscopy was performed at the end of periphyton establishment, at day 30 (Supplementary Fig. 1c). For this, three to five chambers per condition were taken, plastic walls of the chambers were removed with special adaptors and the glass slides were placed in petri dish with 25 mL fresh COMBO medium. Schematic representation of sample preparation for CLSM imaging is shown in Supplementary Fig. 1c. The typical z-range covered 200-300 µm. Live imaging was performed on intact biofilms without fixation or staining.

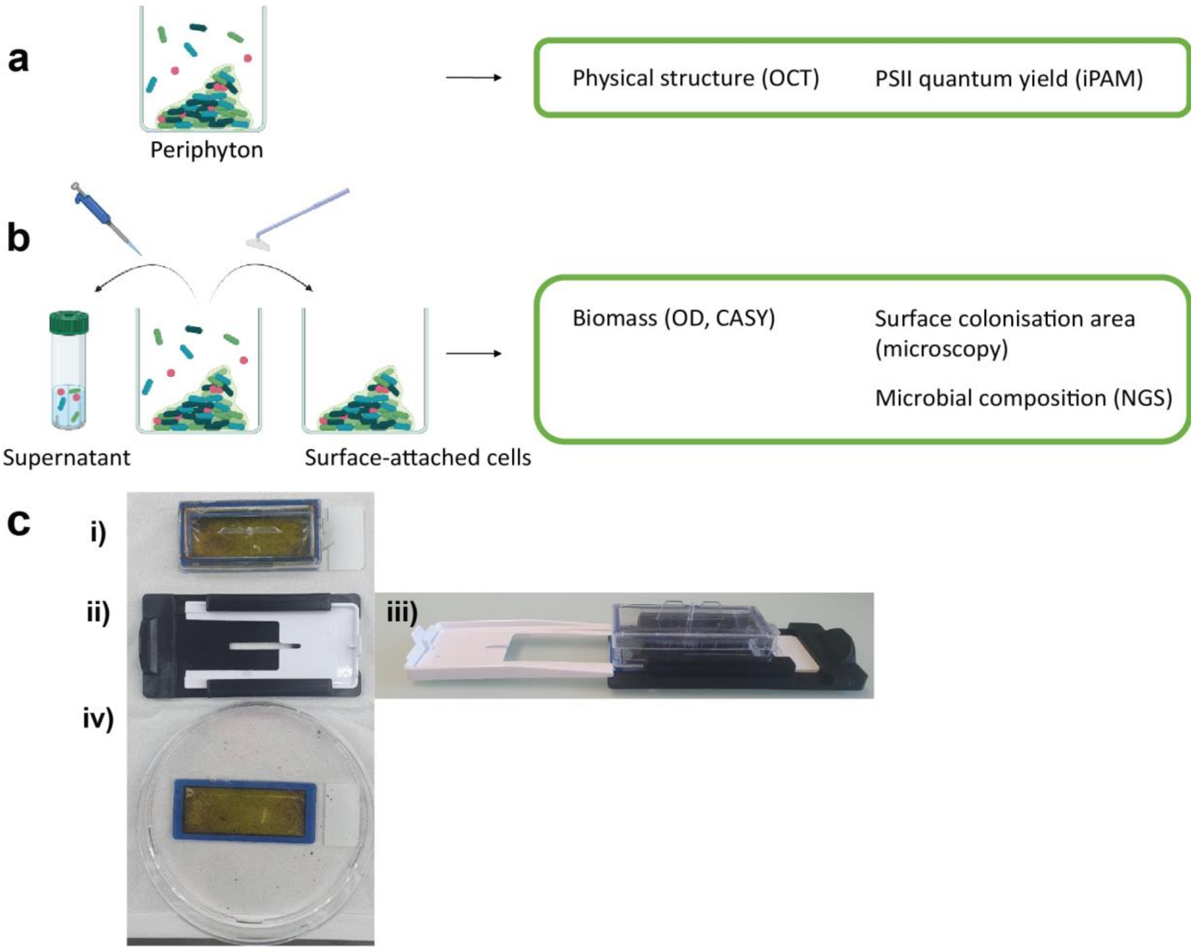

Supplementary Figure 1: Schematic representation of the measurements performed during the establishment of the synthetic periphyton. Physical structure and PSII quantum yield measurements were non-destructive measurements **(a)**. Benthic (surface-attached cells) biomass, surface colonization area and microbial composition of the surface-attached cells were determined after the supernatant was removed **(b)**. Non-benthic (supernatant cells) biomass was also determined **(b)**. Confocal laser scanning microscopy (CLSM) imaging was performed at day 30 (t_3) with the synthetic periphyton (n = 3 replicates) grown in 1-well Nunc™ Lab-Tek™ II Chambers Slides (**c-i**). For this purpose, by using specific adaptors **(c-ii)** the plastic walls of the chambers were removed **(c-iii)** and the glass slide was placed into the sterile petri dish with 25 ml COMBO medium **(c-iv)**. Microscopic observations were performed on at least five different points of one glass slide using upright Leica SP5 confocal laser scanning microscopy (Heidelberg GmbH, Mannheim, Germany) and 40×/0.75 NA objective (Leica, Plan-Apochromat®).

Supplementary Method 2. DNA Extraction, library construction and sequencing

Library construction consisted of a two-step PCR process. The first PCR amplified the 16S rRNA from cyanobacteria, V4-V5 region of the 18S rRNA gene for eukaryotes and *rbcL* gene using two different primer sets with overhang adapters from [1, 2] and [3] respectively (Supplementary Table 8). The first PCR was performed in triplicate for each DNA sample, negative PCR controls, as well as positive PCR controls for 18S rRNA, *rbcL* and 16S rRNA, consisting of mock communities (Supplementary Tables 9-11). The initial conditions of the first PCR, including cycle number, were first determined via quantitative RT-PCR following an internal protocol developed by the Genetic Diversity Center (GDC), Zürich. A minimal cycle number was used to get enough amplicons while limiting PCR-linked bias. Bias was further limited by performing the first amplification in triplicate for each DNA sample. The PCRs were performed in 25-μL volumes with final concentrations of 1x supplied buffer (KAPA HiFi HotStart ReadyMix, Roche, Switzerland) and 0.3 μM of each forward and reverse primer (Supplementary Table 8). In total, 1 μL of extracted DNA was added, ranging in concentration from 2.35 to 143.98 ng/μL. A negative PCR control was carried out in triplicate, by adding 1 μL of PCR grade water instead of DNA sample, as well as positive PCR controls for 16S rRNA, 18S rRNA and *rbcL*, consisting of mock communities (Supplementary Table 6). The PCR program started with 95 °C for 3 min, followed by 18 cycles (16S rRNA), 29 cycles (*rbcL*) or 24 cycles (18S rRNA) consisting of 95 °C for 20 s, 50 °C (16S rRNA), 58 °C (*rbcL*) or 56 °C (18S rRNA) for 15 s, and 72 °C for 15 s. A final extension of 72 °C for 5 min was performed and the PCR products from the three independent reactions for each sample were then pooled and cleaned. Each of the pooled reactions (a total of 75-μL) were cleaned using a 0.8x bead:sample volume ratio of selfmade SPRI beads and separated with a magnetic stand following the protocol of the Agencourt AMPure XP Kit (Beckman Coulter). The cleaned up PCR was stored at -20 °C until further processing.

The second PCR, consisting of a limited-cycle amplification, was carried out to add multiplexing indices and Illumina sequencing adapters. Each sample was dual-indexed by using the Nextera® Index Kit A and D (Illumina, USA). The index PCR was performed in a 20-μL volume with final concentrations of 1x supplied buffer (KAPA HiFi HotStart ReadyMix, Roche, Switzerland) and 0.3 μM of each Nextera Index forward and reverse primer. Two μL of cleaned amplicons from the previous step were added. The PCR program started with 95 °C for 3 min, followed by 10 cycles consisting in 95 °C for 30 s, 55 °C for 30 s, and 72 °C for 30 s. A final extension of 72 °C for 5 min was performed and the PCR products were cleaned as described above.

The DNA concentration of the cleaned and indexed libraries was then determined using a Qubit (1.0) fluorimeter following recommended protocols for the dsDNA HS Assay. The libraries were then normalized and pooled at a 3.2 nM concentration. The pooled libraries were cleaned up twice and DNA concentration determined using a Qubit (1.0) fluorimeter. Absence of adapters was checked with a HS 1000 chip on a TapeStation device (Agilent). PHiX control was added at a 1% concentration. Paired end (2 × 300 nt) sequencing was performed on an Illumina MiSeq (MiSeq Reagent kit v3, 300 cycles) at the Genomic Diversity Centre (GDC) at the ETH, Zurich, Switzerland following the manufacture's run protocols (Illumina, Inc.). The MiSeq Control Software Version 2.2 including MiSeq Reporter 2.2 was used for the primary analysis and the de-multiplexing of the raw reads.

Supplementary Method 3. Script for the ImageJ using custom-written macro and "Huang dark" auto-thresholding

```
//run("Brightness/Contrast...");
setMinAndMax(145, 25000);
setAutoThreshold("Huang dark");
//run("Threshold...");
setThreshold(3500, 65535);
run("Analyze Particles...", "  show=[Count Masks] display summarize");
```

Supplementary Discussion 1. Creation and validation of a specific reference database for single-species detection and quantification in the synthetic periphyton

Because the ability to accurately assign species in a sample is directly related to the quality of the reference database, we first created our own database for the twenty-six single species that were used to establish the synthetic periphyton and their respective amplicon gene sequences (Supplementary Table 5). Briefly, DNA was extracted from single species and genes of interest (18S rRNA, 16S rRNA and *rbcL* genes) were amplified in a PCR reaction (the list of primers is shown in Supplementary Table 7). Then the PCR products were purified and Sanger sequenced (see Methods for more details). Obtained database of single species amplicon sequences was used to assign Amplicon Sequence Variants (ASVs) to single species (Supplementary Table 5). We tested the accuracy of the approach for single species identification and the quantification of their relative abundance by using a mock community composed of a defined mixture of extracted DNA from the twenty-six phototrophic species (Supplementary Table 6).

Sequencing of the 18S rRNA gene gave good resolution at the genus level, allowing detection of all green algae and all diatoms within the mock community (Supplementary Fig. 6a). However, by using these 18S rRNA primers it was not possible to distinguish two diatom species (*Fragilaria crotonensis* and *Fragilaria capucina*) and two green algae species (*Pediastrum duplex* and *Pediastrum boryanum*) of the same genus. We overcame the limitation for *Fragilaria* species by additionally sequencing *rbcL* gene. Note that depending on the used primers for amplicon sequencing, slightly different species abundances were observed in the mock community (Supplementary Fig. 6a and 6b). Although equal DNA amounts from all single species, except for *Cyclotella*, were added into the mock community (Supplementary Table 6), no equal abundances of these species were measured (Supplementary Fig. 6a and 6b). Moreover, not a double amount of *Cyclotella* was measured compared to the other species in the mock community. Together our data point at variations in gene copy numbers and/or differences in PCR primer efficiencies among the species [4]. With 18S rRNA gene primers the lowest abundance was detected for *Tabellaria* sp. (Supplementary Fig. 6a). Godhe *et al.* suggested that diatom cell length and cell biovolume could be a proxy for 18S gene copy number per cell due to a correlation between the cell size and gene copy numbers [4]. However, since the cell biovolume of *Tabellaria* is not the smallest from all species in our community (data not shown), the low abundance of *Tabellaria* probably results from poor PCR amplification efficiency from the DNA of this species rather than cell size.

Plastid large subunit of ribulose-1,5-bisphosphate carboxylase (*rbcL*) is a preferred locus in amplicon sequencing of diatoms [5], since it enables higher taxonomic resolution at the species level and discrimination between species of the same genus. When we used *rbcL* barcode, only diatom species were detected in the mock community, confirming the specificity of the primers (Supplementary Fig. 5b). By using chloroplast-encoded *rbcL* gene, we also measured the highest abundance of *Cyclotella meneghiniana*. This was expected, since double DNA amount of this species was added to the mock community and since *Cyclotella meneghiniana* harbors high chloroplast numbers and thus many *rbcL* genes. However, also with *rbcL* gene barcode, we did not measure equal abundances among the other species in the mock community although equal DNA amounts were used. The lowest abundances were detected for *Nitschia* and *Sellaphora* genera, which could be explained by their small cell biovolume and low chloroplast numbers per cell [6]. Indeed, Vasselon *et al.* showed a correlation between the number of copies of the *rbc*L gene per cell and the diatom biovolume [6]. Our results fit this correlation with the smallest cells (e.g., *Nitzschia* and *Sellaphora)* and largest cells (e.g., *Cymbella*, *Fragilaria* and

Gomphonema) showing the lowest and highest abundances, respectively (Supplementary Fig. 6b).

Among the four cyanobacteria species that we tested, double DNA amount of *Pseudanabaena galeta* was added into the mock community, in order to check the ability to obtain respective community composition with the selected 16S rRNA primers [1]. Our results show that by using 16S rRNA gene as a barcode, approximately double amount of this species compared to other cyanobacteria was measured (Supplementary. Fig. 6c). On the other hand, although equal DNA amount of *Chamaesiphon polonicus*, *Merismopedia glauca* and *Phormidium sp.* was added to the mock community, their relative abundances differed, suggesting variability in 16S rRNA gene copy numbers [7]. Note that we also detected amplification from the chloroplast DNA, which reflects non-specific binding of the used 16S rRNA primers.

In summary, our results show that 18S rRNA gene primers provide resolution sufficient for green algae and diatoms at the genus level, whereas *rbcL* primers enabled higher taxonomic resolution for diatoms, down to the species level. Moreover, the 16S rRNA primers allowed distinguishing the four cyanobacteria species from each other, although an unspecific amplification from the chloroplast was also detected. Depending on the primers used for amplicon sequencing, slightly different single species abundances were observed in the mock community due to variations in gene copy numbers and/or primer specificities. This demonstrates the importance to test for the precision of the selected primers in the amplicon sequencing approach for species quantification before performing community composition analysis. It also shows that targeting more than one gene, even within one phototrophic group, assures accurate community characterization at the species level.

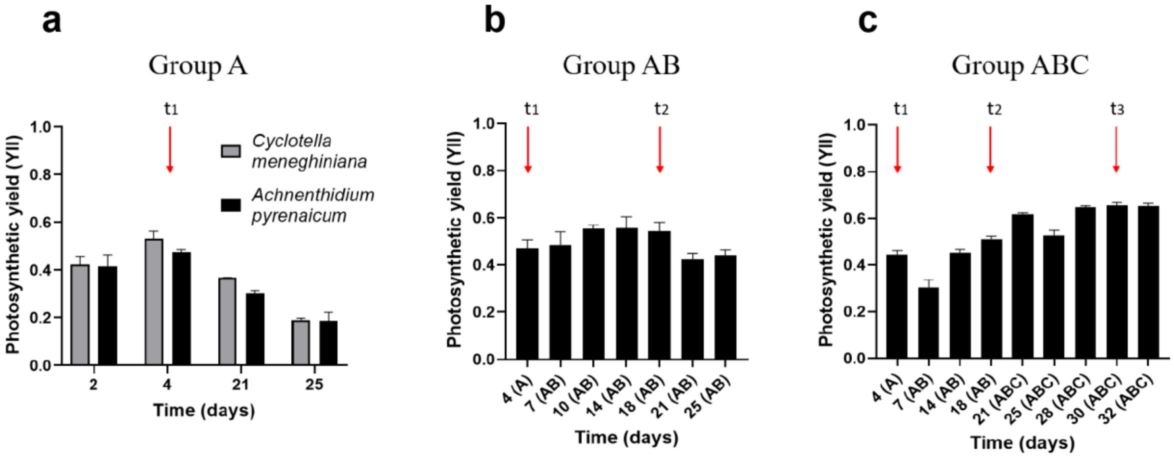

Supplementary Figure 2. Measurement (mean ± s.d, n = 5) of the photosynthetic quantum yield of group A **(a)**, group AB **(b)** and group ABC **(c)** species. Photosynthetic quantum yield was measured by using iPAM (imaging Pulse-Amplitude-Modulation M-series) chlorophyll fluorimeter. Red vertical arrows indicate selected time points when group B species were added on top of group A (t_1), group C on top of group AB (t_2) and periphyton was established (t_3).

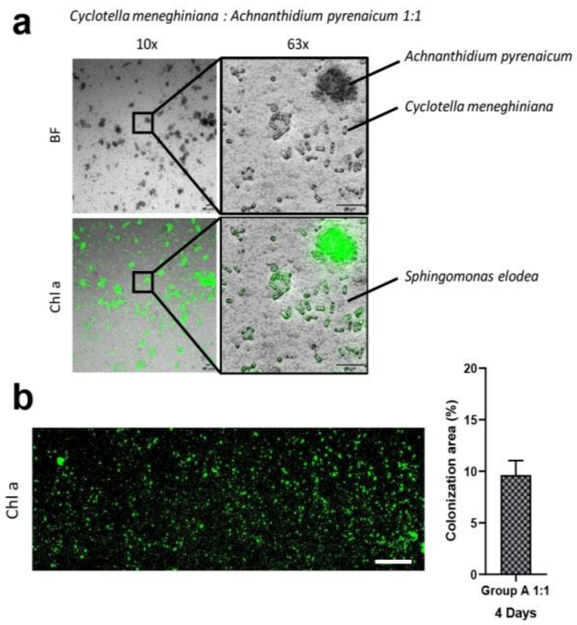

Supplementary Figure 3. Colonization of group A diatom species *Cyclotella meneghiniana* and *Achnanthidium pyrenaicum* together at 1:1 ratio **(a)**. 9 x 3 microscopy images were acquired, montaged and stitched together in order to calculate the percentage of surface colonized area (mean ± s.d, n = 3), (scale bar 1000 µm), (see Methods for more details) **(b)**.

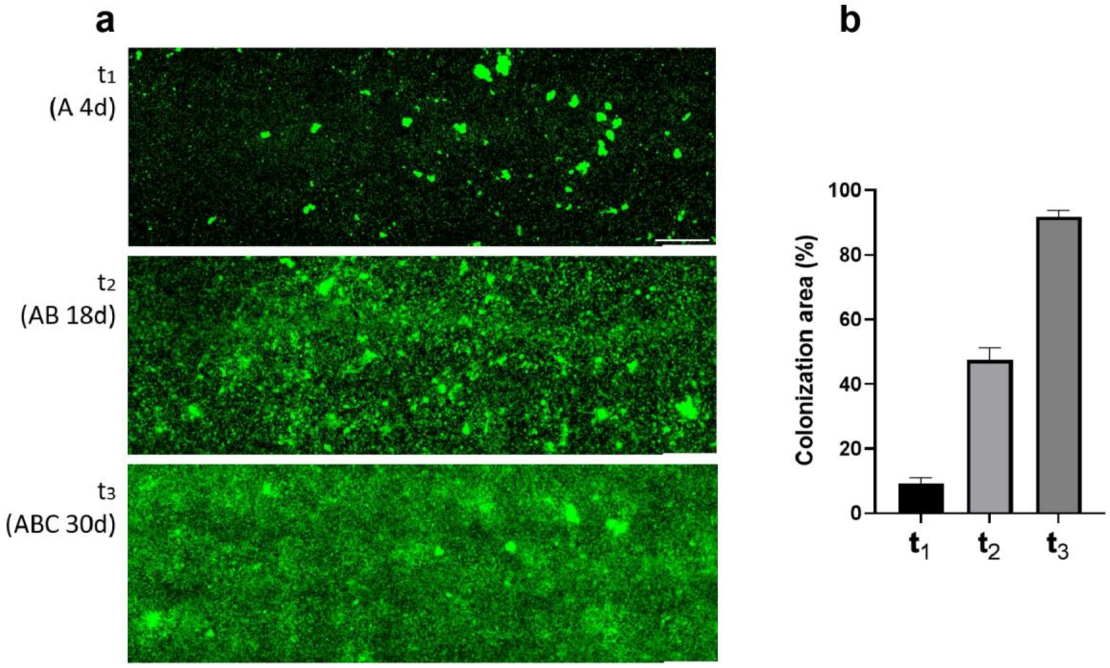

Supplementary Figure 4: Example of representative microscopy images **(a)** showing surface colonization by the phototrophic species during periphyton growth at t_1 (4 days), t_2 (18 days) and t_3 (30 days) (scale bar 1000 µm). Microscopy images (3 replicates from 3 independent experiments) were used to calculate the percentage of surface colonized area (mean ± s.d, n =9) **(b)**.

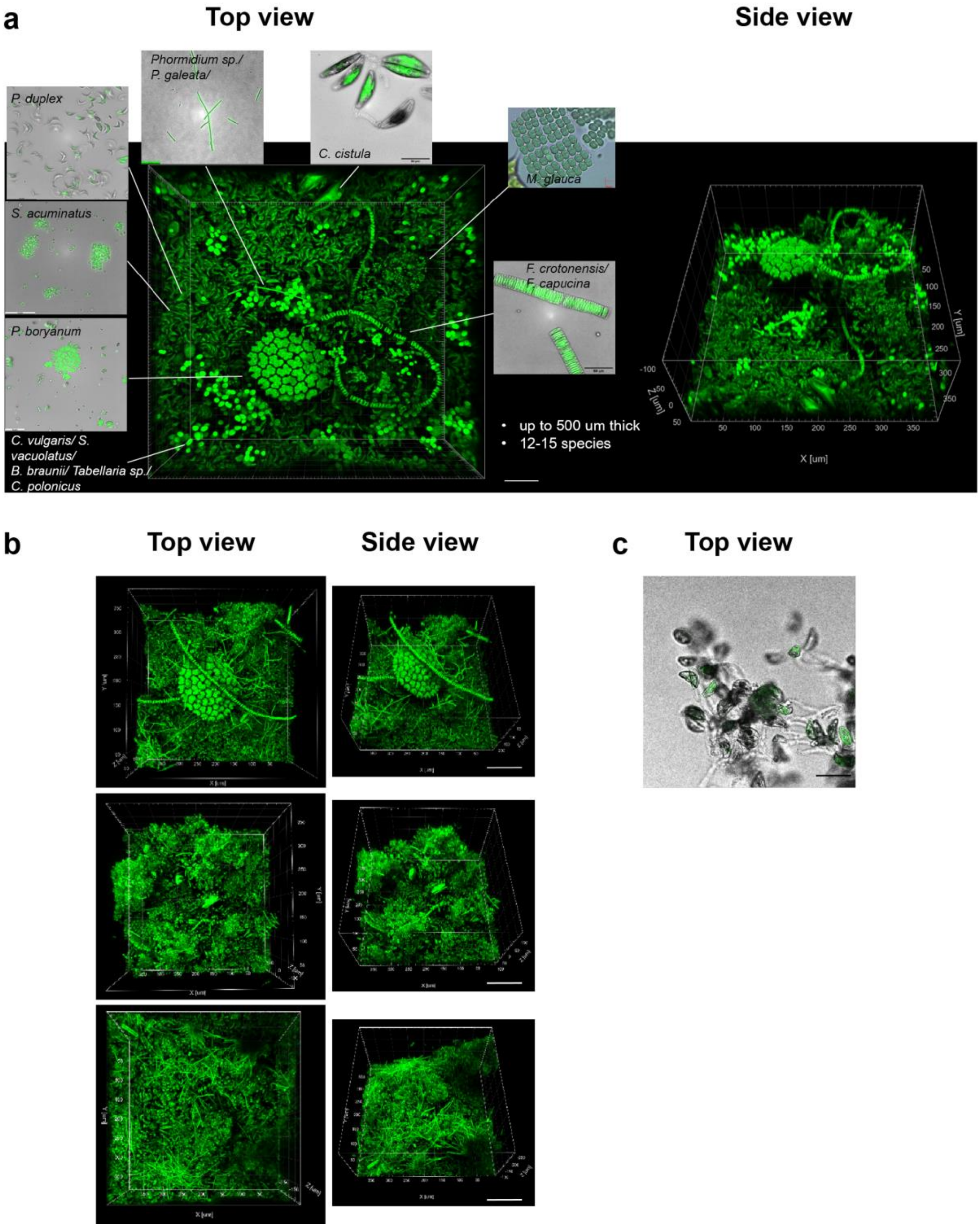

Supplementary Figure 5: Representative confocal laser scanning microscopy images of the established periphyton community at 30 days (t_3) with 12-15 identified phototrophic species based on their morphology (scale bar 50 µm) **(a)**. Additional species (i.e., *C. cuspidata*, *C. accomoda*, *Melosira sp*., *N. vermicularis*, *Synedra sp*. and *Ulnaria ulna*) were also identified from other images (not shown). The green color indicates autofluorescence of chlorophyll *a* (excitation at 633 nm and emission at 650-750 nm). Microscopic observations were performed on at least five different points of one glass slide. Other representative images are shown in **(b)** (scale bar 100 µm). Representative image showing stalks and extracellular polymeric substances produced by species of the community obtained with T-PMT brightfield channel (scale bar 100 µm) **(c)**.

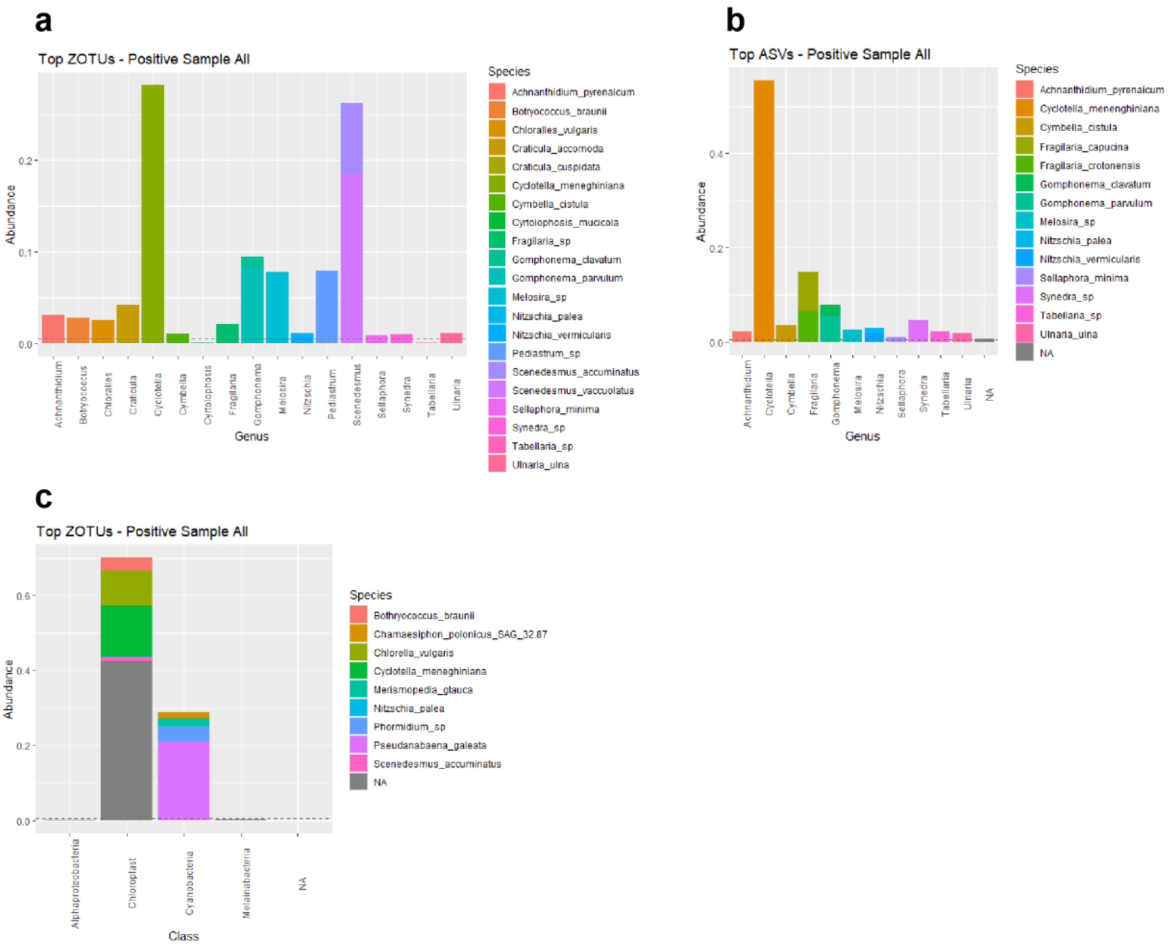

Supplementary Figure 6. Abundances of single phototrophic species in the mock community from taxonomy-based clustering at the genus or class level of assigned 18S rRNA **(a)**, *rbcL* **(b)** and 16S rRNA **(c)** gene sequences. 200 ng DNA from *Cylotella meneghiniana* and *Pseudanabaena galeata* each and 100 ng DNA from each of the 24 remaining single species were pooled into the mock community. NA: not assigned species. Dashed horizontal line represents relative abundance of 0.01.

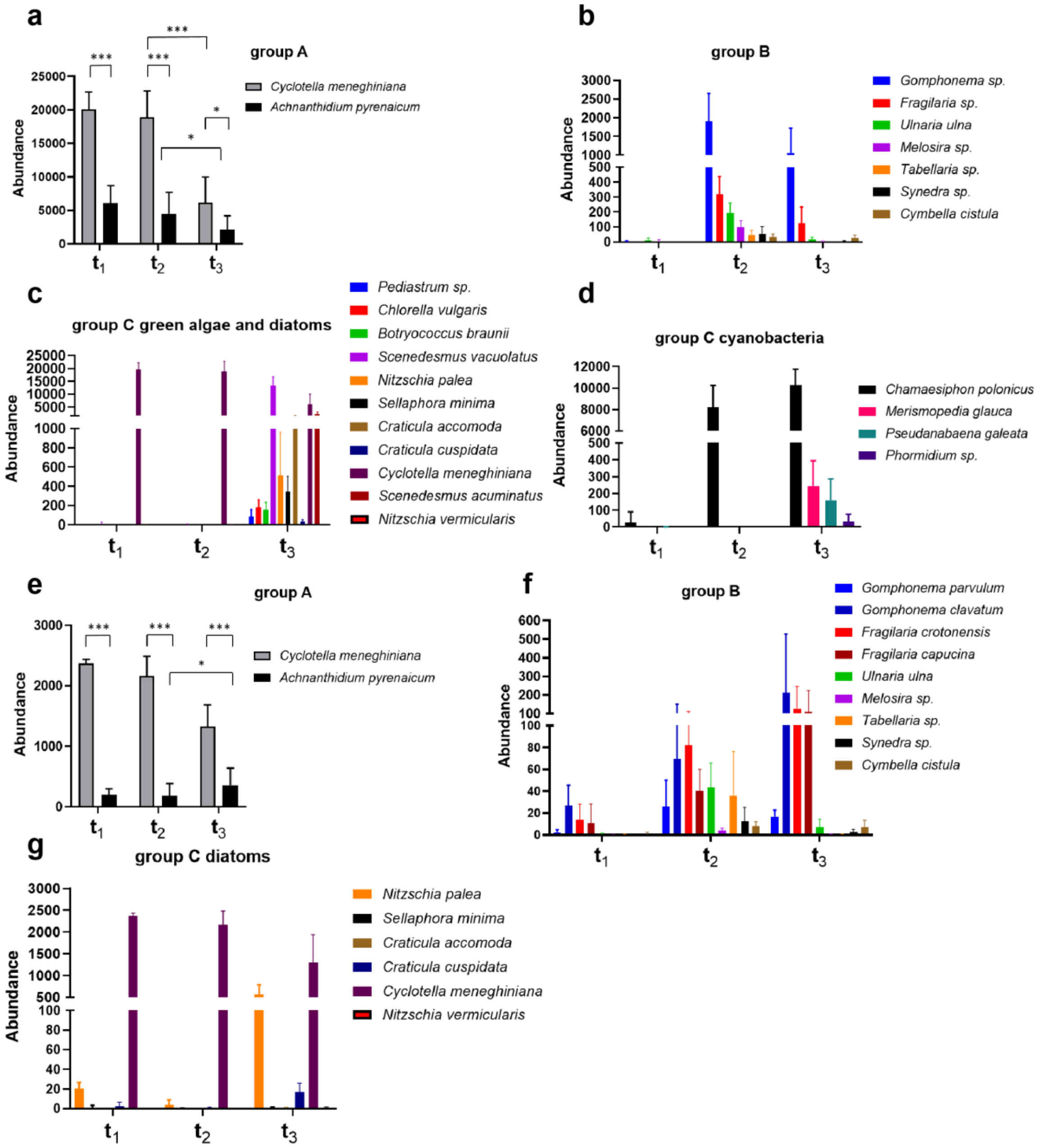

Supplementary Figure 7. Synthetic periphyton composition during periphyton establishment at the species level measured with 18S rRNA (**a-c**), 16S rRNA (**d**) and *rbcL* gene sequencing (**e-g**). Shown is the community composition profile and abundances inferred from taxonomy-based clustering at species or genus level of assigned genes at 4 (t_1), 18 days (t_2) and 30 days (t_3) during periphyton formation (mean ± s.d, n = 9). Abundances of group A **(a)**, group B **(b)** and group C **(c)** eukaryotic species (green algae and diatoms) measured using 18S rRNA amplicon sequence were measured at t_1, t_2 and t_3 during periphyton establishment. Abundances of group C cyanobacteria **(d)** were measured using 16S rRNA. Finally, abundances of group A **(e)**, group B **(f)** and group C **(g)** diatom species were measured with *rbcL*. ns – not significant, * P<0.05, ** P<0.005 *** P<0.0001, one-way ANOVA test followed by Tukey's test. Using *rbcL*, we detected more than two group A species at t_1, pointing at non-specific binding of *rbcL* primers. However, due to low concentrations of extracted DNA from timepoint t_1 and since the overall abundance of non-specific counts was less than 5% of the community, these counts were treated as noise.

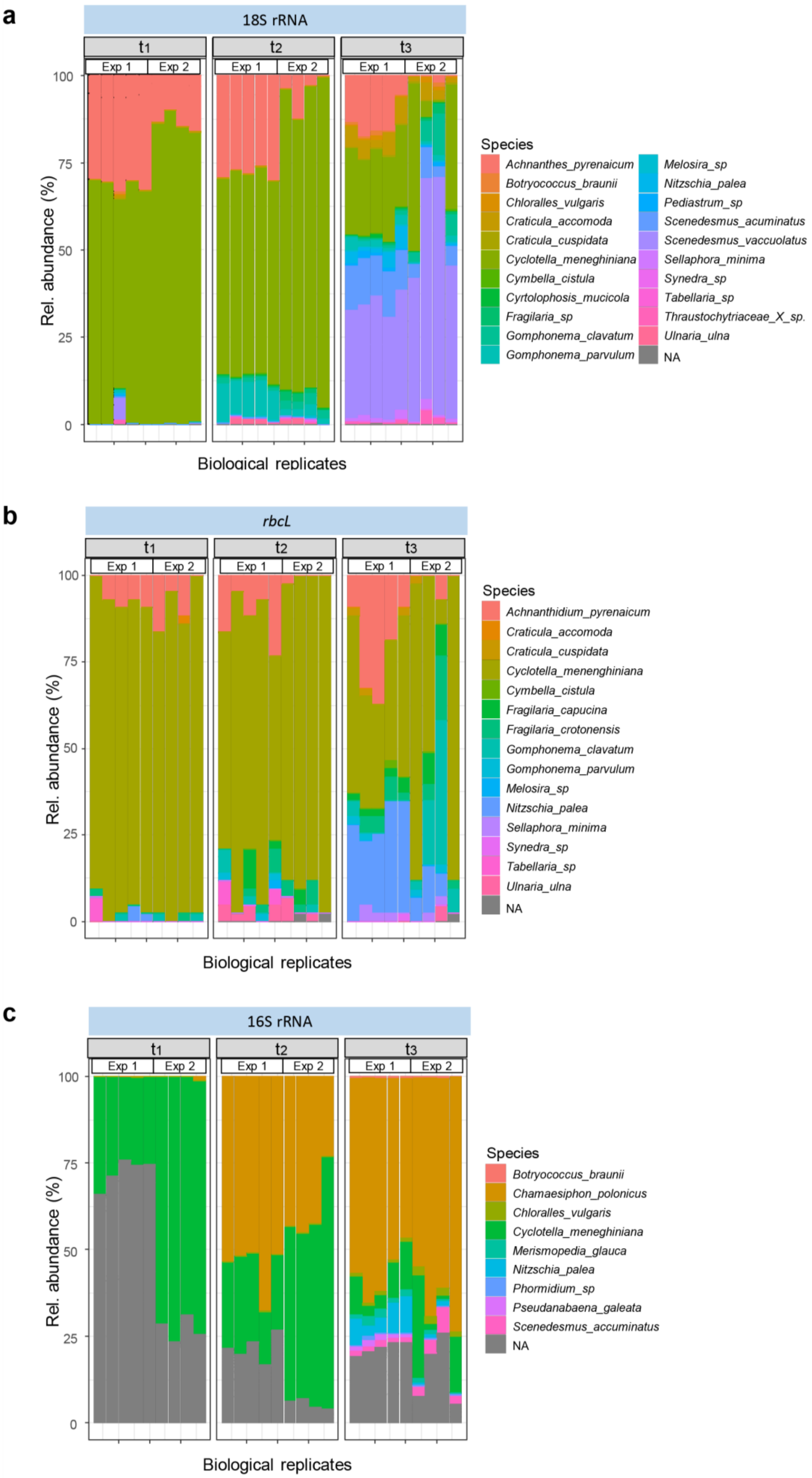
a
18S rRNA
t1
t2
t3
Exp 1
Exp 2
100
75
50
25
0
Rel. abundance (%)
Biological replicates
Species
Achnanthes_pyrenaicum
Botryococcus_braunii
Chloralles_vulgaris
Craticula_accomoda
Craticula_cuspidata
Cyclotella_meneghiniana
Cymbella_cistula
Cyrtolophosis_mucicola
Fragilaria_sp
Gomphonema_clavatum
Gomphonema_parvulum
Melosira_sp
Nitzschia_palea
Pediastrum_sp
Scenedesmus_acuminatus
Scenedesmus_vaccuolatus
Sellaphora_minima
Synedra_sp
Tabellaria_sp
Thraustochytriaceae_X_sp.
Ulnaria_ulna
NA
b
rbcL
Species
Achnanthidium_pyrenaicum
Craticula_accomoda
Craticula_cuspidata
Cyclotella_menenghiniana
Cymbella_cistula
Fragilaria_capucina
Fragilaria_crotonensis
Gomphonema_clavatum
Gomphonema_parvulum
Melosira_sp
Nitzschia_palea
Sellaphora_minima
Synedra_sp
Tabellaria_sp
Ulnaria_ulna
NA
c
16S rRNA
Species
Botryococcus_braunii
Chamaesiphon_polonicus
Chloralles_vulgaris
Cyclotella_meneghiniana
Merismopedia_glauca
Nitzschia_palea
Phormidium_sp
Pseudanabaena_galeata
Scenedesmus_accuminatus
NA

Supplementary Figure 8. Reproducibility of the community composition during periphyton establishment. Data are obtained from two independent experiments (Exp 1 and Exp 2, with n = 5 and 4 biological replicates, respectively). Profile and relative abundances were inferred from taxonomy-based clustering at species level of assigned 18S rRNA **(a)**, *rbcL* **(b)** and 16S rRNA **(c)** genes at 4 (t_1), 18 (t_2) and 30 days (t_3) during periphyton formation. NA: not assigned species.

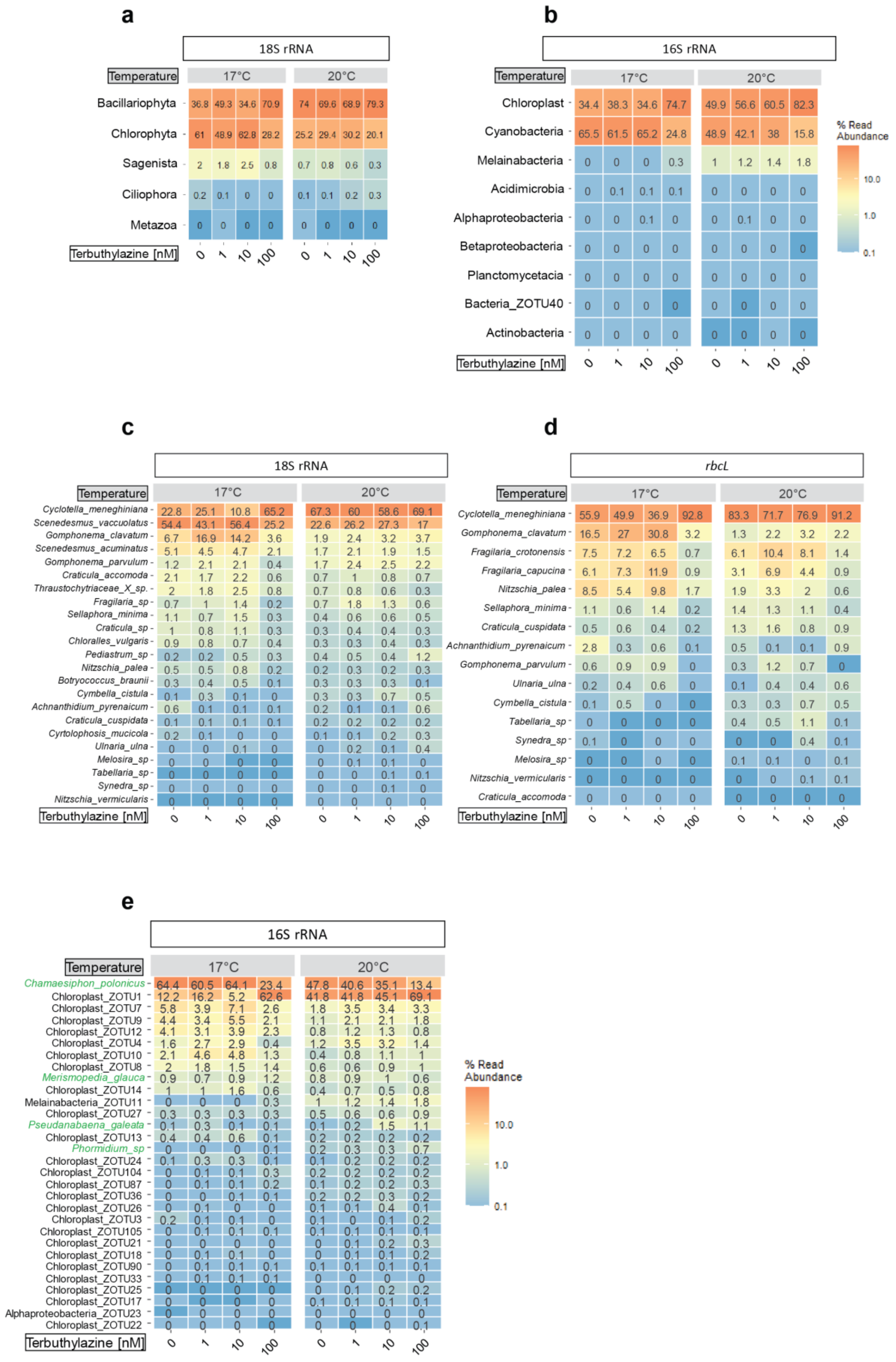

a
18S rRNA
Temperature
17°C
20°C
Bacillariophyta
Chlorophyta
Sagenista
Ciliophora
Metazoa
Terbuthylazine [nM]
b
16S rRNA
Chloroplast
Cyanobacteria
Melainabacteria
Acidimicrobia
Alphaproteobacteria
Betaproteobacteria
Planctomycetacia
Bacteria_ZOTU40
Actinobacteria
% Read Abundance
c
Cyclotella_meneghiniana
Scenedesmus_vaccuolatus
Gomphonema_clavatum
Scenedesmus_acuminatus
Gomphonema_parvulum
Craticula_accomoda
Thraustochytriaceae_X_sp.
Fragilaria_sp
Sellaphora_minima
Craticula_sp
Chloralles_vulgaris
Pediastrum_sp
Nitzschia_palea
Botryococcus_braunii
Cymbella_cistula
Achnanthidium_pyrenaicum
Craticula_cuspidata
Cyrtolophosis_mucicola
Ulnaria_ulna
Melosira_sp
Tabellaria_sp
Synedra_sp
Nitzschia_vermicularis
d
rbcL
Fragilaria_crotonensis
Fragilaria_capucina
Ulnaria_ulna
Tabellaria_sp
e
Chamaesiphon_polonicus
Merismopedia_glauca
Melainabacteria_ZOTU11
Pseudanabaena_galeata
Phormidium_sp
Alphaproteobacteria_ZOTU23

Supplementary Figure 9. Heatmaps showing relative abundances of the phototrophic species in established periphyton under different conditions. Effects of terbuthylazine (0, 1, 10 or 100 nM) and temperature (17 °C or 20 °C) are shown at t_3 (ABC 30 days) at the phylum level measured with 18S rRNA **(a)** and 16S rRNA **(b)** and at the species level measured with 18S rRNA **(c)**, *rbcL* **(d)** and 16S rRNA **(e)** genes. Species labelled in green correspond to the 4 cyanobacteria that we used to establish the periphyton. Data are based on the average of Amplicon Sequence Variants (ASVs) abundance (n = 3-4).

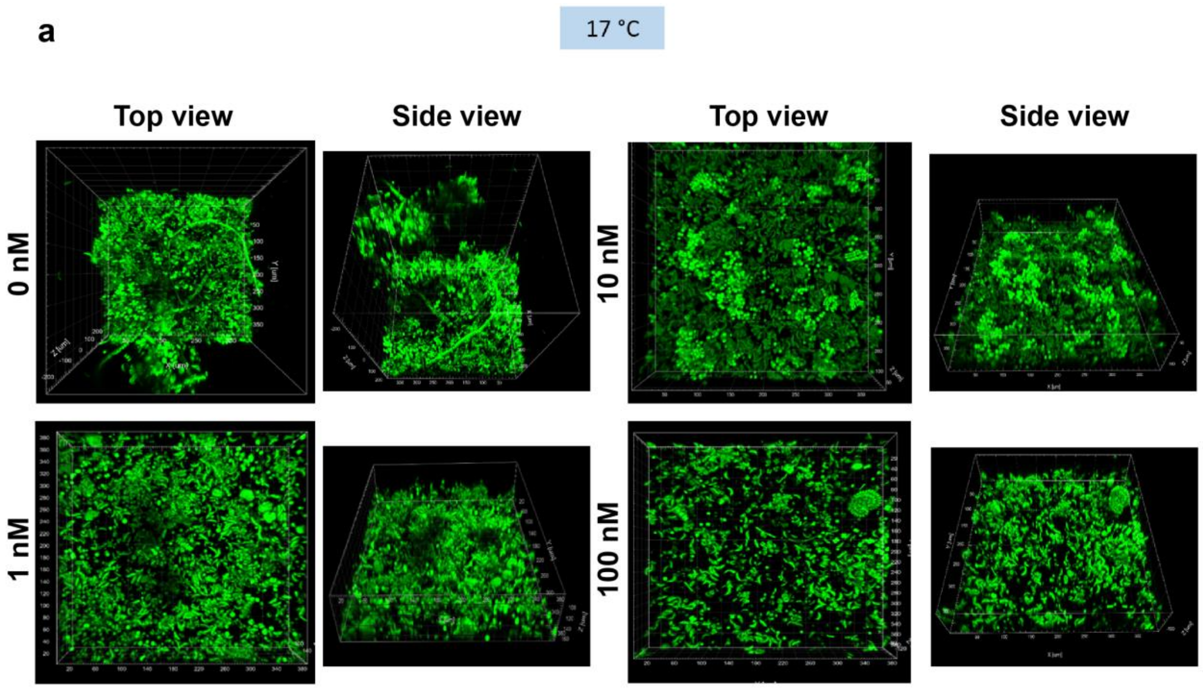

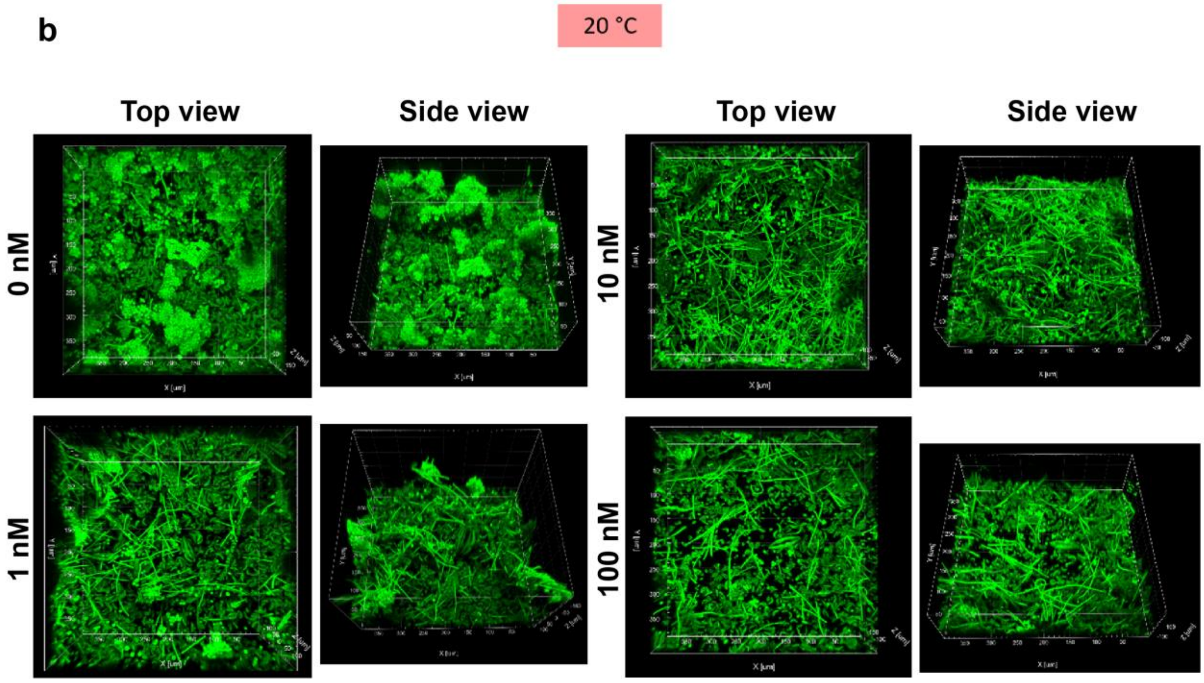

Supplementary Figure 10: Representative confocal laser scanning microscopy images of established periphyton community at 30 days (t_3) under different treatments. The treatments correspond to 4 levels of the herbicide terbuthylazine (0 1, 10 and 100 nM) and 2 different temperatures 17 °C **(a)** and 20 °C **(b)**. The green color indicates autofluorescence of chlorophyll *a* (excitation at 633 nm and emission at 650-750 nm).

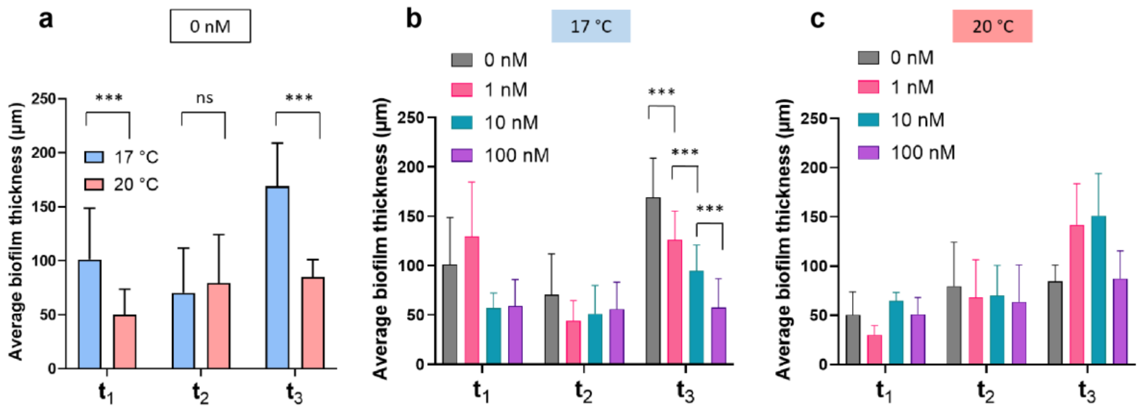

Supplementary Figure 11: Average biofilm thickness of periphyton grown under different conditions. OCT measurements were obtained over 30 days of periphyton growth at time points t_1 (A 4 days), t_2 (AB 18 days) and t_3 (ABC 30 days). Average biofilm thickness of periphyton grown under no terbuthylazine exposure at 17°C or 20°C is shown in **(a)**. The conditions correspond to periphyton grown in the presence of 0 nM (control), 1 nM, 10 nM or 100 nM terbuthylazine and at 17 °C **(b)** or 20 °C **(c)**. All experiments were performed in triplicate (mean ± s.d, n = 3), average biofilm thickness was determined from 20-30 images. ns – not significant, * P<0.05, ** P<0.005 *** P<0.0001, based on Students t-test for (A) and post hoc Tukey's test for **(b)** and **(c)**.

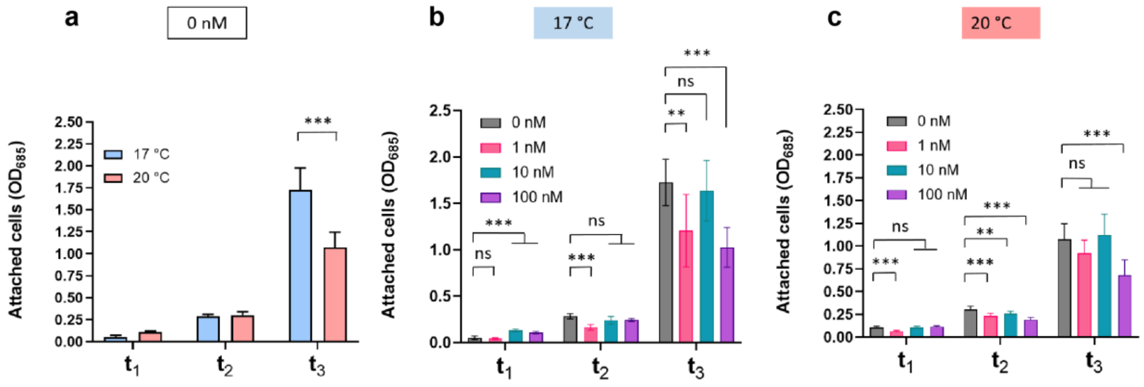

Supplementary Figure 12: Benthic biomass of periphyton grown under different conditions. Attached cells were measured with the spectrophotometer at 685 nm over 30 days of periphyton growth at time points t_1 (A 4 days), t_2 (AB 18 days) and t_3 (ABC 30 days). Benthic biomass of periphyton grown under no terbuthylazine exposure at 17°C (blue) or 20°C (red) is shown in **(a)**. Benthic biomass of periphyton grown in the presence of 0 nM (control), 1 nM, 10 nM or 100 nM terbuthylazine and at 17 °C **(b)** or 20 °C **(c)** are also shown. All experiments were performed in triplicate (mean ± s.d, n = 3). ns – not significant, * P<0.05, ** P<0.005 *** P<0.0001, based on Students t-test for **(a)** and post hoc Tukey's test for **(b)** and **(c)**.

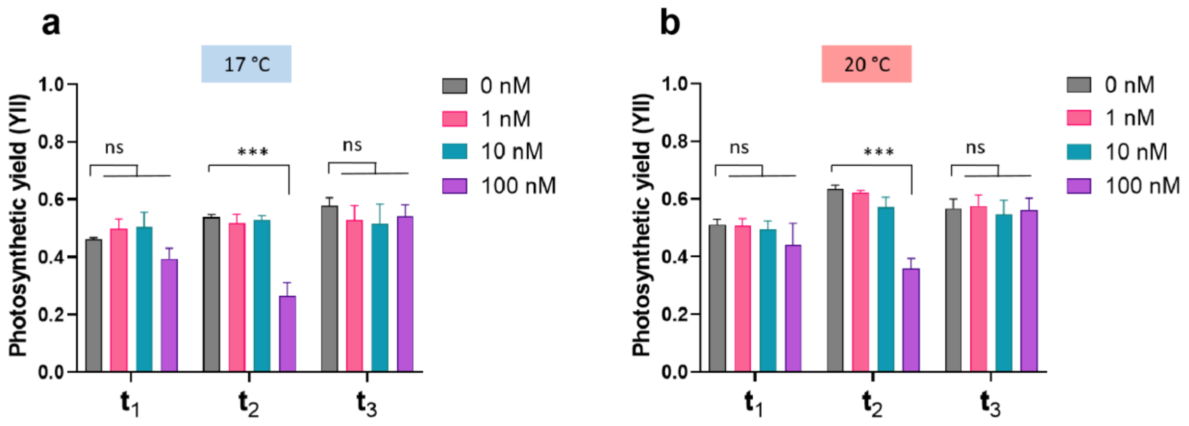

Supplementary Figure 13. Photosynthetic yield of periphyton grown under different conditions. Photosynthetic efficiency measurements were obtained over 30 days of periphyton growth at time points t_1 (A 4 days), t_2 (AB 18 days) and t_3 (ABC 30 days). The conditions correspond to periphyton grown in the presence of 0 nM (control), 1 nM, 10 nM or 100 nM terbuthylazine and at 17 °C **(a)** or 20 °C **(b)**. Photosynthetic yield ($\Delta F/F_m'$) was measured as described in 2.3.3. Material and Methods. All experiments were performed in 5 replicates (mean ± s.d, n = 5). ns – not significant, * P<0.05, ** P<0.005 *** P<0.0001, based on post hoc Tukey's test.

Supplementary Table 1. The initially selected 34 phototrophic microbial species for the establishment of the synthetic periphyton.

Phylum	Genus and species names	Algothek Nr.[1]	Ecological guild[2]	Group
**Bacillariophyceae**	*Achnanthidium pyrenaicum*	TCC832	Low profile	A
	Cyclotella meneghiniana	SAG 1020-1a	Low profile/ planktonic	A and C
	Cocconeis placentula var. euglypta	TCC720	Low profile	A
	Gomphonema parvulum	SAG 1032-1	High profile	B
	Gomphonema clavatum	TCC527	High profile	B
	Synedra sp.	CCAC 5476 B	High profile	B
	Fragilaria crotonensis	SAG 28.96	High profile	B
	Fragilaria capucina	CCAC 2678 B	High profile	B
	Melosira sp.	CCAC 1935 B	High profile	B
	Cymbella cistula	CCAC 2680 B	High profile	B
	Tabellaria sp.	CCAC 3717 B	High profile	B
	Ulnaria ulna	TCC634	High profile	B
	Nitzschia palea	SAG 1052-3a	Motile	C
	Nitzschia vermicularis	CCAC 3463 B	Motile	C
	Craticula accomoda (Navicula accomoda)	TCC107	Motile	C
	Sellaphora minima	TCC524	Motile	C
	Craticula cuspidata (Navicula cuspidata	CCAC 3270 B	Motile	C
	Surirella sp	CCAC 3461 B	Motile	C
**Chlorophyceae**	*Mougeotia sp.*	SAG 11.96		C
	Stigeoclonium aestivale	SAG 477-20		C
	Scenedesmus acuminatus	SAG 38.81		C
	Scenedesmus vacuolatus	SAG 211-8b		C
	Oedogonium sp.	SAG 54.94		C
	Chlorella vulgaris	SAG 211-8m		C
	Ulothrix mucosa	SAG 56.9		C
	Botryococcus braunii	SAG 30.81		C
	Pediastrum diplex	SAG 84.80		C
	Pediastrum boryanum	SAG 87.81		C
**Cyanobacteria**	*Chamaesiphon polonicus*	SAG 32.87		C
	Lyngbya sp.	SAG 36.91		C
	Merismopedia glauca	SAG 48.79		C
	Pseudanabaena galeata	SAG 13.83		C
	Phormidium sp.	SAG 37.9		C
	Anabaena sp.	SAG 12.82		C

[1] SAG: Culture collection of algae, Goettingen university, Germany; CCAC: Central collection of algae, Duisburg-Essen university, Germany; TCC: Thonon culture collection, INRAE, France

[2] S. I. Passy in Diatom ecological guilds display distinct and predictable behavior along nutrient and disturbance gradients in running waters, Vol. 86 Elsevier, 2007, pp. 171-178. DOI: 10.1016/j.aquabot.2006.09.018

Low profile guild diatoms: the most resistant species with regard to nutrient-poor conditions, and therefore dominate at low nutrient levels.

High profile guild diatoms: have long, filamentous, chain-forming, stalked morphology or comparatively small cells that form long colonies and extend beyond the boundary layer, which allows them to exploit resources that are unavailable to the low profile guild species.
Motile guild diatoms: moving species

Supplementary Table 2. Composition of the COMBO medium. Six major compounds, except $NaHCO_3$, were mixed together and autoclaved. After cooling, $NaHCO_3$, algal trace elements and vitamins were added under sterile conditions.

Compound	Stock (g l-1)	Stock concentration (mol l-1)	Final concentration (µmol l-1)
**Seven major compounds**			
$CaCl_2$ $2H_2O$	36.76	0.5	250
$MgSO_4$ $7H_2O$	36.97	0.15	150
K_2HPO_4	8.71	0.05	50
$NaNO_3$	85.01	1	1000
$NaHCO_3$	12.6	0.24	150
Na_2SiO_3 9H2O	28.42	0.05	100
H_3BO_3	24	0.5	388
KCl	7.45	0.1	100
**Algal trace elements (ATE)**			
Na_2EDTA	4.36	0.11	11.7
$FeCl_3$ x $6H_2O$	1	0.3	3.7
$CuSO_4$ x $5H_2O$		0.04	0.004
$ZnSO_4$ x $7H_2O$		0.05	0.08
$CoCl_2$ x $6H_2O$		0.05	0.05
$MnCl_2$ x $4H_2O$		0.05	0.9
Na_2MoO_4 x $2H_2O$		0.08	0.09
H_2SeO_3		0.05	0.012
Na_3VO_4		0.05	0.01
**Vitamins**			
Thiamine	0.2		0.3
Biotine		0.00004	0.002
Vitamin B12		0.00008	0.0004
HEPES		0.05	25

Supplementary Table 3. Results of the single species planktonic growth measured with spetrophotometer at OD 685 nm. *k* is the growth constant and Ym is the proxy for the reached maximum biomass determined by fitting the growth data into a logistic growth model. The robustness of the fit is shown with R squared. CI = confidence interval at 5%. nd = not determined due the high variability among replicates. Color code: green – fast growth and high biomass; yellow – fast growth and low/intermediate biomass; red – slow growth and low biomass.

Group	Species	Phylum	Growth rate (*k* constant)	CI (*k*)	Maximum reached biomass (Ym)	CI (Ym)	Degrees of Freedom	R squared	Replicates (N)
A	*Achnanthidium pyrenaicum*	Bacillariophyta	0.01655	0.009929 - 0.02662	0.1612	0.1344 - 0.2330	33	0.8216	6
	Cyclotella meneghiniana	Bacillariophyta	0.0177	0.01688 - 0.01854	0.2848	0.2810 - 0.2888	45	0.9962	6
B	*Gomphonema parvulum*	Bacillariophyta	0.01106	0.008404 - 0.01395	0.2316	0.1716 - 0.5231	33	0.9668	6
	Gomphonema clavatum	Bacillariophyta	0.01417	0.01260 - 0.01584	0.2108	0.1921 - 0.2386	33	0.9919	6
	Synedra sp.	Bacillariophyta	0.01447	0.01255 - 0.01655	0.1953	0.1819 - 0.2134	33	0.9809	6
	Fragilaria crotonensis	Bacillariophyta	0.01542	0.01326 - 0.01780	0.06838	0.06589 - 0.07117	45	0.9353	6
	Fragilaria capucina	Bacillariophyta	0.03336	0.02556 - 0.04495	0.04797	0.04534 - 0.05069	39	0.9027	6
	Melosira sp.	Bacillariophyta	0.01854	0.01493 - 0.02303	0.05072	0.04823 - 0.05366	39	0.9417	6
	Cymbella cistula	Bacillariophyta	0.01819	0.01478 - 0.02212	0.03451	0.03261 - 0.03681	51	0.9144	9
	Tabellaria sp.	Bacillariophyta	0.007536	0.005651 - 0.009457	0.142	0.09432 - 0.8594	33	0.9709	6
	Ulnaria ulna	Bacillariophyta	0.01514	0.01006 - 0.02131	0.04297	0.03763 - 0.05356	80	0.7182	6
C	*Scenedesmus acuminatus*	Chlorophyta	0.01891	0.01315 - 0.02571	0.7972	0.6633 - 1.289	5	0.9958	3
	Chlorella vulgaris	Chlorophyta	0.008993	nd	0.73511	nd	5	0.9625	3
	Pediastrum boryanum	Chlorophyta	0.01099	0.005007 - 0.01871	0.3933	nd	5	0.9811	3
	Merismopedia glauca	Cyanobacteria	0.008799	0.004858 - 0.01335	0.747	0.5219 - 14.25	48	0.8288	6
	Cyclotella meneghiniana	Bacillariophyta	0.0177	0.01688 - 0.01854	0.2848	0.2810 - 0.2888	45	0.9962	6
	Nitzschia palea	Bacillariophyta	0.02462	0.02260 - 0.02684	0.1967	0.1920 - 0.2016	33	0.9917	6
	Nitzschia vermicularis	Bacillariophyta	0.01255	0.001036 - 0.03907	0.02292	nd	39	0.2169	6
	Craticula cuspidata	Bacillariophyta	0.007552	0.004742 - 0.01038	0.06568	0.04803 - 0.4322	30	0.9386	6
	Sellaphora (Navicula) minima	Bacillariophyta	0.02505	0.01425 - 0.05194	0.1978	0.1668 - 0.2558	33	0.8224	6
	Scenedesmus vacuolatus	Chlorophyta	0.01703	0.009682 - 0.02617	0.5362	0.4379 - 1.139	5	0.9894	3
	Pediastrum duplex	Chlorophyta	0.02138	0.009093 - 0.04111	0.6605	0.5427 - 1.266	5	0.9673	3
	Chamaesiphon polonicus	Cyanobacteria	0.00686	nd	0.5259	nd	59	0.7371	6
	Phormidium sp.	Cyanobacteria	0.02221	nd	0.295	nd	48	0.6184	6
	Pseudanabaena galeata	Cyanobacteria	0.0199	0.01701 - 0.02314	0.5205	0.4803 - 0.5744	21	0.9868	6
	Botryococcus braunii	Chlorophyta	0.005539	0.003867 - 0.01026	0.4169	0.1510 - ???	21	0.9261	3
	Craticula accomoda	Bacillariophyta	0.02244	0.01945 - 0.02583	0.2027	0.1960 - 0.2097	44	0.9706	6

Supplementary Table 4. Results of the single species planktonic growth measured by using an automated CASY cell counter Model TT (Roche Innovatis AG). *k* is the growth constant and Ym is the proxy for the reached maximum biomass determined by fitting the growth data into a logistic growth model. The robustness of the fit is shown with R squared. CI = confidence interval at 5%. nd = not determined due the high variability among replicates. Color code: green – fast growth and high biomass; yellow – fast growth and low/intermediate biomass; red – slow growth and low biomass.

Group	Species	Phylum	Growth rate (*k* constant)	CI (*k*)	Maximum reached biomass (Ym)	CI (Ym)	Degrees of Freedom	R squared	Replicates (N)
A	*Achnanthidium pyrenaicum*	Bacillariophyta	0.01665	0.01030 to 0.02628	5.59E+05	471031 to 786155	30	0.8609	6
	Cyclotella meneghiniana	Bacillariophyta	0.04031	0.03242 to 0.05023	9.49E+05	921248 to 978591	30	0.9603	6
B	*Gomphonema parvulum*	Bacillariophyta	0.01607	0.01008 to 0.02418	8.00E+05	671170 to 1159746	27	0.8925	6
	Gomphonema clavatum	Bacillariophyta	0.01311	0.006895 to 0.02106	7.20E+05	540364 to 2254174	29	0.8022	6
	Synedra sp.	Bacillariophyta	0.01536	0.01157 to 0.01998	9.96E+05	872827 to 1234110	30	0.9483	6
	Fragilaria crotonensis	Bacillariophyta	0.09141	nd	2.10E+05	191491 to 227962	24	0.8325	6
	Fragilaria capucina	Bacillariophyta	0.02361	nd	1.18E+06	nd	15	0.6841	3
	Melosira sp.	Bacillariophyta	0.01229	0.008747 to 0.01643	3.09E+05	273874 to 373758	36	0.8838	6
	Cymbella cistula	Bacillariophyta	not measured						
	Tabellaria sp.	Bacillariophyta	0.01443	0.008561 to 0.02196	1.73E+05	143656 to 243763	27	0.826	6
	Ulnaria ulna	Bacillariophyta	0.01143	nd	2.15E+05	nd	26	0.6215	6
C	*Scenedesmus acuminatus*	Chlorophyta	0.01094	nd	nd	nd	18	0.9934	3
	Chlorella vulgaris	Chlorophyta	0.01529	0.01245 to 0.01844	4.35E+06	3944243 to 5120158	18	0.9906	3
	Pediastrum boryanum	Chlorophyta	not measured						
	Merismopedia glauca	Cyanobacteria	not measured						
	Cyclotella meneghiniana	Bacillariophyta	0.04031	0.03242 to 0.05023	9.49E+05	921248 to 978591	30	0.9603	6

Group	Species	Phylum	Growth rate (*k* constant)	CI (*k*)	Maximum reached biomass (Ym)	CI (Ym)	Degrees of Freedom	R squared	Replicates (N)
**C**	*Nitzschia palea*	Bacillariophyta	0.06934	nd	1.03E+06	980259 to 1085575	30	0.8991	6
	Nitzschia vermicularis	Bacillariophyta	0.01223	0.005389 to 0.02166	1.75E+05	126721 to 495549	27	0.6566	6
	Craticula cuspidata	Bacillariophyta	0.02151	0.01191 to 0.04166	7.75E+05	664270 to 956435	30	0.747	6
	Sellaphora (Navicula) minima	Bacillariophyta	0.01886	0.01231 to 0.02894	1.32E+06	1118804 to 1770488	30	0.8998	6
	Scenedesmus vacuolatus	Chlorophyta	0.02163	0.01731 to 0.02680	2.44E+06	2291976 to 2625606	21	0.9774	3
	Pediastrum duplex	Chlorophyta	not measured							
	Chamaesiphon polonicus	Cyanobacteria	not measured							
	Phormidium sp.	Cyanobacteria	not measured							
	Pseudanabaena galeata	Cyanobacteria	not measured							
	Botryococcus braunii	Chlorophyta	not measured							
	Craticula accomoda	Bacillariophyta	0.01385	0.006458 to 0.02839	1.99E+05	160055 to 351045	27	0.7586	6

Supplementary Table 5. Reference database for 26 the single species of the community and their corresponding sequences of 18S rRNA gene, *rbcL* and 16S rRNA, used for species identification in the community with next generation sequencing.

Phylum	Gene used for molecular identification	18S rRNA				
	Genus and species names	Accession no.	Best match in Blast analysis (accession no.)	Similarity BLAST (%)	ASV (18S rRNA)	Similarity Sanger (%)*
**Green algae**	*Scenedesmus acuminatus*	OL304135	AB917119.1	100	ZOTU8	90
	Scenedesmus vacuolatus	OL304136	MH651242.1	99.82	ZOTU2, ZOTU3	90
	Chlorella vulgaris	OL304137	MN248531.1	99.83	ZOTU21, ZOTU27	89
	Botryococcus braunii	OL304138	JF261275.2	99.82	ZOTU24	90
	Pediastrum duplex	OL304156	AY780662.1	100	ZOTU17	90
	Pediastrum boryanum	OL304139	HM021307.1	99.65	ZOTU17	96
**Diatoms**	*Gomphonema parvulum*	OL304140	KT072961.1	100	ZOTU7, ZOTU51, ZOTU39, ZOTU63, ZOTU92	89
	Nitzschia palea	OL304141	MN696721.1	99.82	ZOTU16	89
	Fragilaria crotonensis	OL304142	MG022768.1	99.48	ZOTU10, ZOTU13	90
	Cyclotella meneghiniana	OL304143	HQ912576.1	99.82	ZOTU1, ZOTU58, ZOTU65	90
	Craticula cuspidata	OL304144	KM999000.1	100	ZOTU29, ZOTU33, ZOTU34, ZOTU35, ZOTU23, ZOTU31	90
	Cymbella cistula	OL304145	AM502017.1	98.41	ZOTU19, ZOTU88, ZOTU69	88
	Fragilaria capucina	OL304146	OL304146.1	100	ZOTU10, ZOTU13	90
	Melosira sp.	OL304147	AY569589.1	99.64	ZOTU18	90
	Nitzschia vermicularis	OL304148	AJ867279.1	95.36	ZOTU30, ZOTU80	89
	Synedra sp.	OL304149	KF959661.1	100	ZOTU22, ZOTU26	90
	Tabellaria sp.	OL304150	MH356258.1	99.83	ZOTU25	90
	Achnanthidium pyrenaicum	OL304151	KY863466.1	100	ZOTU4, ZOTU9, ZOTU59, ZOTU67, ZOTU37, ZOTU50, ZOTU42, ZOTU43, ZOTU56, ZOTU91	89
	Craticula accomoda	OL304152	KF959652.1	99.65	ZOTU12, ZOTU33, ZOTU34, ZOTU35, ZOTU23, ZOTU31	90
	Sellaphora (Navicula) minima	OL304153	KF959656.1	100	ZOTU14	89
	Ulnaria ulna	OL304154	AM497727.1	100	ZOTU11	78
	Gomphonema clavatum	OL304155	KC736622.1	99.82	ZOTU6, ZOTU15, ZOTU40, ZOTU86, ZOTU57, ZOTU36, ZOTU55, ZOTU78, ZOTU45	88

* maximum likelihood of ASV to Sanger sequence

Phylum	Gene used for molecular identification	*rbcL*				
	Genus and species names	Accession no.	Best match in Blast analysis (accession no.)	Similarity BLAST (%)	ASV (*rbcL*)	Similarity Sanger (%)*
**Diatoms**	*Gomphonema parvulum*	OK669058	JQ003567.1	98.06	ZOTU10, ZOTU32, ZOTU29	89
	Nitzschia palea	OK669059	KJ542520.1	98.39	ZOTU3	89
	Fragilaria crotonensis	OK669060	KF959640.1	98.39	ZOTU4, ZOTU8, ZOTU58	89
	Cyclotella meneghiniana	OK669061	JQ003565.1	98.07	ZOTU1, ZOTU33, ZOTU47, ZOTU51, ZOTU53, ZOTU59	89
	Craticula cuspidata	OK669062	KM999070.1	98.39	ZOTU13, ZOTU56, ZOTU41, ZOTU48	89
	Cymbella cistula	OK669063	KJ011808.1	98.07	ZOTU14, ZOTU43, ZOTU55	89
	Fragilaria capucina	OK669064	KF959640.1	98.06	ZOTU5	89
	Melosira sp.	OK669065	HQ912470.1	91.26	ZOTU16, ZOTU27, ZOTU22, ZOTU26	89
	Nitzschia vermicularis	OK669066	MN734080.1	91.94	ZOTU21, ZOTU30, ZOTU31, ZOTU35	89
	Synedra sp.	OK669067	JQ003569.1	96.32	ZOTU9, ZOTU17	89
	Tabellaria sp.	OK669068	HQ912448.1	95.82	ZOTU11, ZOTU18, ZOTU19, ZOTU60	89
	Achnanthidium pyrenaicum	OK669069	KY799135.1	98.35	ZOTU6, ZOTU42, ZOTU54	89
	Craticula accomoda	OK669070	LC335884.1	90.97	ZOTU49, ZOTU23	89
	Sellaphora (Navicula) minima	OK669071	KF959642.1	98.34	ZOTU12, ZOTU20, ZOTU40, ZOTU57	89
	Ulnaria ulna	OK669072	HQ912454.1	90.14	ZOTU7, ZOTU46, ZOTU15, ZOTU52	89
	Gomphonema clavatum	OK669073	LR742612.1	99.04	ZOTU2, ZOTU39, ZOTU24, ZOTU34	89

Phylum	Gene used for molecular identification	16S rRNA				
	Genus and species names	Accession no.	Best match in Blast analysis (accession no.)	Similarity BLAST (%)	ASV (16S rRNA)	Similarity Sanger (%)*
**Cyanobacteria**	*Chamaesiphon polonicus*	OK737797	KY704111.1	99.76	ZOTU2	93
	Merismopedia glauca	OK737798	HF678499.1	99.53	ZOTU15	93
	Pseudanabaena galeata	OK737799	LC016778.1	99.75	ZOTU5	92
	Phormidium sp.	OK737800	AM398795.1	99.76	TOTU16	93

* maximum likelihood of ASV to Sanger sequence

Supplementary Table 6. Species composition in the Mock communities used as positive controls in the library construction for the 18S rRNA, *rbcL* and 16S rRNA gene sequencing. The positive control consisted of a mixture of DNA from 16 diatom, 6 green algae and 4 cyanobacterial species for 18S rRNA, *rbcL* and 16S rRNA genes, respectively. Additionally, a mixture of DNA from 10 bacteria in a Mock N°4 from the Genetic Diversity Centre in Zürich was used.

Target	Positive control	Genus and species names	Reference
18S rRNA gene/ rbcL/ 16S rRNA	mock community of all species	*Achnanthidium pyrenaicum*	This study
		*Cyclotella meneghiniana **	
		Gomphonema parvulum	
		Gomphonema clavatum	
		Synedra sp.	
		Fragilaria crotonensis	
		Fragilaria cf. Capucina	
		Melosira sp.	
		Cymbella cistula	
		Tabellaria sp.	
		Ulnaria ulna	
		Nitzschia palea	
		Nitzschia vermicularis	
		Navicula accomoda (Craticula accomoda)	
		Sellaphora minima	
		Navicula cuspidata (Craticula cuspidata)	
		Scenedesmus acuminatus	
		Scenedesmus vacuolatus	
		Chlorella vulgaris	
		Botryococcus braunii	
		Pediastrum duplex	
		Pediastrum boryanum	
		Chamaesiphon polonicus	
		Merismopedia glauca	
		*Pseudanabaena galeata **	
		Phormidium sp.	
		Anabaena sp.	
	mock community of 16 diatoms	*Achnanthidium pyrenaicum*	
		*Cyclotella meneghiniana **	
		Gomphonema parvulum	
		Gomphonema clavatum	
		Synedra sp.	
		Fragilaria crotonensis	
		Fragilaria cf. Capucina	
		Melosira sp.	
		Cymbella cistula	

Target	Positive control	Genus and species names	Reference
18S rRNA gene/ *rbcL* / 16S rRNA	mock community of 16 diatoms	*Tabellaria sp.*	This study
		Ulnaria ulna	
		Nitzschia palea	
		Nitzschia vermicularis	
		Navicula accomoda (Craticula accomoda)	
		Sellaphora minima	
		Navicula cuspidata (Craticula cuspidata)	
	mock community of 6 green algae species	*Scenedesmus acuminatus*	
		Scenedesmus vacuolatus	
		Chlorella vulgaris	
		Botryococcus braunii	
		Pediastrum duplex	
		Pediastrum boryanum	
	mock community of 5 cyanobacteria species	*Chamaesiphon polonicus*	
		Merismopedia glauca	
		Pseudanabaena galeata *	
		Phormidium sp.	
16S rRNA gene	16S	*Pseudarthrobacter chlorophenolicus*	Mock N°4 from the GDC Zürich**
		Bacillus subtilis	
		Burkholderia xenovorans	
		Escherichia coli	
		Micrococcus luteus	
		Paenibacillus sabinae	
		Pseudomonas protogens	
		Pseudomonas stutzeri	
		Streptomyces violaceoruber	
		Xanthobacter autotrophicus	

* mock community of synthetic peripyton. 100 ng of DNA from all single species, except *Cyclotella meneghiniana* and *Pseudanabaena galeata* of which 200 ng DNA each, were mixed.

** Genetic Diversity Center, ETH Zürich, Switzerland. https://gdc.ethz.ch/

Supplementary Table 7. Primers used in this study for PCR amplification of 18S, 16S rRNA and *rbcL* libraries.

Region of interest	Primer name	Sequence (5'-3')	Ta	Reference
V4-V5 (18S rRNA gene)	574*f (forward)<br>1132r (reverse)	CGGTAAYTCCAGCTCYV<br>CCGTCAATTHCTTYAART	56 °C	2
16S rRNA	CYA359F (forward)<br>CYA781R (reverse)	GGGGAATYTTCCGCAATGGG<br>GACTACWGGGGTATCTAATCCCWTT	50 °C	1
rbcL	Diat_rbcL_708F (forward)<br>R3 (reverse)	AGGTGAARYWAAAGGTTCWTACTTAAA<br>CCTTCTAATTTACCWACWACWG	56 °C	3

Supplementary Table 8. Primers used in this study for the construction of 18S, 16S rRNA and *rbcL* libraries. For each primer, four variants were created by inserting a different number of bases (0 to 3) between the region of interest and the adapter. These insertions create more heterogeneity on the flow cell. For each primer, an equimolar pool of the four variants was used for the first PCR.

Region of interest	Primer name	Primer variant name	Sequence (5'-3')	Ref.
V4-V5 (18S rRNA gene)	574*f (forward)	574*f_nex0	TCGTCGGCAGCGTCAGATGTGTATAAGAGACAGGACGGTAAYTCCAGCTCYV	2
		574*f_nex1	TCGTCGGCAGCGTCAGATGTGTATAAGAGACAGNGACGGTAAYTCCAGCTCYV	
		574*f_nex2	TCGTCGGCAGCGTCAGATGTGTATAAGAGACAGNNGACGGTAAYTCCAGCTCYV	
		574*f_nex3	TCGTCGGCAGCGTCAGATGTGTATAAGAGACAGNNNGACGGTAAYTCCAGCTCYV	
	1132r (reverse)	1132r_nex0	GTCTCGTGGGCTCGGAGATGTGTATAAGAGACAGCACCGTCAATTHCTTYAART	2
		1132r_nex1	GTCTCGTGGGCTCGGAGATGTGTATAAGAGACAGNCACCGTCAATTHCTTYAART	
		1132r_nex2	GTCTCGTGGGCTCGGAGATGTGTATAAGAGACAGNNCACCGTCAATTHCTTYAART	
		1132r_nex3	GTCTCGTGGGCTCGGAGATGTGTATAAGAGACAGNNNCACCGTCAATTHCTTYAART	
rbcL	Diat_rbcL_708F	708F_nex0	TCGTCGGCAGCGTCAGATGTGTATAAGAGACAGGAGGTGAARYWAAAGGTTCWTACTTAAA	3
		708F_nex1	TCGTCGGCAGCGTCAGATGTGTATAAGAGACAGNGAGGTGAARYWAAAGGTTCWTACTTAAA	
		708F_nex2	TCGTCGGCAGCGTCAGATGTGTATAAGAGACAGNNGAGGTGAARYWAAAGGTTCWTACTTAAA	
		708F_nex3	TCGTCGGCAGCGTCAGATGTGTATAAGAGACAGNNNGAGGTGAARYWAAAGGTTCWTACTTAAA	
	R3 (reverse)	R3_nex0	GTCTCGTGGGCTCGGAGATGTGTATAAGAGACAGTCCTTCTAATTTACCWACWACWG	3
		R3_nex1	GTCTCGTGGGCTCGGAGATGTGTATAAGAGACAGNTCCTTCTAATTTACCWACWACWG	
		R3_nex2	GTCTCGTGGGCTCGGAGATGTGTATAAGAGACAGNNTCCTTCTAATTTACCWACWACWG	
		R3_nex3	GTCTCGTGGGCTCGGAGATGTGTATAAGAGACAGNNNTCCTTCTAATTTACCWACWACWG	
16S rRNA	CYA359F (forward)	CYA359F_nex0	TCGTCGGCAGCGTCAGATGTGTATAAGAGACAGG GGGGAATYTTCCGCAATGGG	1
		CYA359F_nex1	TCGTCGGCAGCGTCAGATGTGTATAAGAGACAGNGGGGGAATYTTCCGCAATGGG	
		CYA359F_nex2	TCGTCGGCAGCGTCAGATGTGTATAAGAGACAGNNGGGGGAATYTTCCGCAATGGG	
		CYA359F_nex3	TCGTCGGCAGCGTCAGATGTGTATAAGAGACAGNNNGGGGGAATYTTCCGCAATGGG	
	CYA781R (reverse)	CYA781R_nex0	GTCTCGTGGGCTCGGAGATGTGTATAAGAGACAGAGACTACWGGGGTATCTAATCCCWTT	1
		CYA781R_nex1	GTCTCGTGGGCTCGGAGATGTGTATAAGAGACAGNAGACTACWGGGGTATCTAATCCCWTT	
		CYA781R_nex2	GTCTCGTGGGCTCGGAGATGTGTATAAGAGACAGNNAGACTACWGGGGTATCTAATCCCWTT	
		CYA781R_nex3	GTCTCGTGGGCTCGGAGATGTGTATAAGAGACAGNNNAGACTACWGGGGTATCTAATCCCWTT	

Supplementary Table 9. Read processing reports for 18S rRNA gene. Read counts for each sample for each of the processing steps. The mean length of the reads is also indicated in the table. The composition of mock communities used as positive controls is described in SI Table 5. 0 nM, 1 nM, 10 nM and 100 nM correspond to 0 nM (unexposed control), 1 nM, 10 nM and 100 nM terbuthylezine exposure, respectively.

							Read counts				
Target	Sample	Experiment	Timepoint	Temperature (°C)	Terbuthylazine (nM)	Replicate	Raw	Merged	Primer	Clean	Mean read length (bp)
**18S rRNA gene**	C001	Periphyton establishment	t1 (4 days)	17	-	R01	68676	68676	67592	66218	276
	C002	Periphyton establishment	t1 (4 days)	17	-	R02	54415	54415	53571	53474	276
	C003	Periphyton establishment	t1 (4 days)	17	-	R03	540	540	527	521	275
	C004	Periphyton establishment	t1 (4 days)	17	-	R04	80934	80934	79649	79537	276
	C005	Periphyton establishment	t1 (4 days)	17	-	R05	61260	61260	60342	60274	276
	C006	Periphyton establishment	t2 (18 days)	17	-	R01	56844	56844	55996	55920	276
	C007	Periphyton establishment	t2 (18 days)	17	-	R02	49870	49870	49102	49052	276
	C008	Periphyton establishment	t2 (18 days)	17	-	R03	48980	48980	48247	48186	276
	C009	Periphyton establishment	t2 (18 days)	17	-	R04	38462	38462	37914	37852	276
	C010	Periphyton establishment	t2 (18 days)	17	-	R05	43307	43307	42668	42621	276
	C011	Periphyton establishment	t3 (30 days)	17	-	R01	39595	39595	39079	39008	275
	C012	Periphyton establishment	t3 (30 days)	17	-	R02	32835	32835	32365	32312	275
	C013	Periphyton establishment	t3 (30 days)	17	-	R03	44592	44592	43976	43909	275
	C014	Periphyton establishment	t3 (30 days)	17	-	R04	37474	37474	36965	36907	275
	C015	Periphyton establishment	t3 (30 days)	17	-	R05	42154	42154	41541	41486	275
	C016	Case study	t1 (4 days)	17	0 nM	R01	27737	27737	27297	27268	276
	C017	Case study	t1 (4 days)	17	0 nM	R02	53659	53659	52857	52776	276
	C018	Case study	t1 (4 days)	17	0 nM	R03	49053	49053	48294	48247	276
	C019	Case study	t1 (4 days)	17	0 nM	R04	86830	86830	85515	85389	276
	C020	Case study	t1 (4 days)	17	1 nM	R01	51857	51857	50881	50798	276
	C021	Case study	t1 (4 days)	17	1 nM	R02	54339	54339	53523	53456	276
	C022	Case study	t1 (4 days)	17	1 nM	R03	77414	77414	76164	76059	276
	C023	Case study	t1 (4 days)	17	10 nM	R01	48490	48490	47788	47723	276
	C024	Case study	t1 (4 days)	17	10 nM	R02	41843	41843	41180	41139	276
	C025	Case study	t1 (4 days)	17	10 nM	R03	64281	64281	63237	63092	276
	C026	Case study	t1 (4 days)	17	100 nM	R01	52507	52507	51777	51721	276
	C027	Case study	t1 (4 days)	17	100 nM	R02	42776	42776	42175	42130	276
	C028	Case study	t1 (4 days)	17	100 nM	R03	40865	40865	40286	40235	276
	C029	Case study	t2 (18 days)	17	0 nM	R01	58802	58802	57985	57925	276
	C030	Case study	t2 (18 days)	17	0 nM	R02	56833	56833	56004	55944	276
	C031	Case study	t2 (18 days)	17	0 nM	R03	59475	59475	58694	58632	276
	C032	Case study	t2 (18 days)	17	0 nM	R04	55750	55750	55013	54960	276
	C033	Case study	t2 (18 days)	17	1 nM	R01	55019	55019	54254	54172	276
	C034	Case study	t2 (18 days)	17	1 nM	R02	54495	54495	53695	53638	276
	C035	Case study	t2 (18 days)	17	1 nM	R03	51947	51947	51211	51131	276
	C036	Case study	t2 (18 days)	17	10 nM	R01	45234	45234	44549	44501	276

Target	Sample	Experiment	Timepoint	Temperature (°C)	Terbuthylazine (nM)	Replicate	Read counts Raw	Merged	Primer	Clean	Mean read length (bp)
**18S rRNA gene**	C037	Case study	t2 (18 days)	17	10 nM	R02	54326	54326	53559	53498	276
	C038	Case study	t2 (18 days)	17	10 nM	R03	58685	58685	57859	57793	276
	C039	Case study	t2 (18 days)	17	100 nM	R01	57187	57187	56336	56274	276
	C040	Case study	t2 (18 days)	17	100 nM	R02	64593	64593	63687	63623	276
	C041	Case study	t2 (18 days)	17	100 nM	R03	61725	61725	60881	60791	276
	C042	Case study	t3 (30 days)	17	0 nM	R01	55540	55540	54773	54709	275
	C043	Case study	t3 (30 days)	17	0 nM	R02	51408	51408	50627	50557	275
	C044	Case study	t3 (30 days)	17	0 nM	R03	44460	44460	43784	43718	275
	C045	Case study	t3 (30 days)	17	0 nM	R04	55034	55034	54246	54156	275
	C046	Case study	t3 (30 days)	17	1 nM	R01	51905	51905	51143	51059	275
	C047	Case study	t3 (30 days)	17	1 nM	R02	55229	55229	54494	54438	275
	C048	Case study	t3 (30 days)	17	1 nM	R03	48546	48546	47863	47797	275
	C049	Case study	t3 (30 days)	17	10 nM	R01	53575	53575	52785	52665	275
	C050	Case study	t3 (30 days)	17	10 nM	R02	58059	58059	57124	57056	275
	C051	Case study	t3 (30 days)	17	10 nM	R03	59208	59208	58340	58252	275
	C052	Case study	t3 (30 days)	17	100 nM	R01	56003	56003	55182	55096	275
	C053	Case study	t3 (30 days)	17	100 nM	R02	66879	66879	66014	65924	275
	C054	Case study	t3 (30 days)	17	100 nM	R03	64499	64499	63567	63496	275
	C055	Case study	t1 (4 days)	20	0 nM	R01	48716	48716	48003	47955	276
	C056	Case study	t1 (4 days)	20	0 nM	R02	49580	49580	48850	48784	276
	C057	Case study	t1 (4 days)	20	0 nM	R03	41702	41702	41064	41026	276
	C058	Case study	t1 (4 days)	20	1 nM	R01	42427	42427	41779	41737	276
	C059	Case study	t1 (4 days)	20	1 nM	R02	55311	55311	54466	54410	276
	C060	Case study	t1 (4 days)	20	1 nM	R03	34194	34194	33660	33620	276
	C061	Case study	t1 (4 days)	20	10 nM	R01	34623	34623	34151	34116	276
	C062	Case study	t1 (4 days)	20	10 nM	R02	36092	36092	35539	35503	276
	C063	Case study	t1 (4 days)	20	10 nM	R03	32766	32766	32296	32250	276
	C064	Case study	t1 (4 days)	20	100 nM	R01	37836	37836	37285	37236	276
	C065	Case study	t1 (4 days)	20	100 nM	R02	38417	38417	37835	37772	276
	C066	Case study	t1 (4 days)	20	100 nM	R03	34595	34595	34010	33974	276
	C067	Case study	t2 (18 days)	20	0 nM	R01	55587	55587	54790	54729	276
	C068	Case study	t2 (18 days)	20	0 nM	R02	52252	52252	51549	51502	276
	C069	Case study	t2 (18 days)	20	0 nM	R03	61224	61224	60348	60288	276
	C070	Case study	t2 (18 days)	20	1 nM	R01	56107	56107	55293	55210	276
	C071	Case study	t2 (18 days)	20	1 nM	R02	59535	59535	58680	58619	276
	C072	Case study	t2 (18 days)	20	1 nM	R03	54844	54844	54042	53981	276
	C073	Case study	t2 (18 days)	20	10 nM	R01	61492	61492	60628	60556	276
	C074	Case study	t2 (18 days)	20	10 nM	R02	56686	56686	55854	55794	276
	C075	Case study	t2 (18 days)	20	10 nM	R03	55878	55878	55133	55078	276
	C076	Case study	t2 (18 days)	20	100 nM	R01	47497	47497	46795	46747	276
	C077	Case study	t2 (18 days)	20	100 nM	R02	62183	62183	61262	61195	276

							Read counts				
Target	Sample	Experiment	Timepoint	Temperature (°C)	Terbuthylazine (nM)	Replicate	Raw	Merged	Primer	Clean	Mean read length (bp)
**18S rRNA gene**	C078	Case study	t2 (18 days)	20	100 nM	R03	54213	54213	53475	53424	276
	C079	Case study	t3 (30 days)	20	0 nM	R01	61445	61445	60592	60516	275
	C080	Case study	t3 (30 days)	20	0 nM	R02	56064	56064	55301	55229	275
	C081	Case study	t3 (30 days)	20	0 nM	R03	61135	61135	60276	60191	275
	C082	Case study	t3 (30 days)	20	1 nM	R01	58164	58164	57336	57270	275
	C083	Case study	t3 (30 days)	20	1 nM	R02	58720	58720	57840	57752	275
	C084	Case study	t3 (30 days)	20	1 nM	R03	49973	49973	49227	49149	275
	C085	Case study	t3 (30 days)	20	10 nM	R01	62056	62056	61179	61107	275
	C086	Case study	t3 (30 days)	20	10 nM	R02	57734	57734	56877	56794	275
	C087	Case study	t3 (30 days)	20	10 nM	R03	57622	57622	56847	56781	275
	C088	Case study	t3 (30 days)	20	100 nM	R01	55556	55556	54742	54674	275
	C089	Case study	t3 (30 days)	20	100 nM	R02	59339	59339	58516	58436	275
	C090	Case study	t3 (30 days)	20	100 nM	R03	66661	66661	65762	65677	275
	NegPCR1a	negative control					1806	1806	1519	1290	275
	Pos-mock4	mock4 community					554	554	521	206	274
	Pos-pool-cyano	mock community of 4 cyanobacteria species					59135	59135	58330	58240	275
	Pos-pool-chloro	mock community of 6 green algae species					69703	69703	68719	68618	275
	Pos-pool-diatom	mock community of 16 diatom species					93845	93845	91527	91351	275
	Pos-pool-all	mock community of all species					55291	55291	54529	54463	275

Supplementary Table 10. Read processing reports for *rbcL* gene. Read counts for each sample for each of the processing steps. The mean length of the reads is also indicated in the table. The composition of mock communities used as positive controls is described in SI Table 5. 0 nM, 1 nM, 10 nM and 100 nM correspond to 0 nM (unexposed control), 1 nM, 10 nM and 100 nM terbuthylezine exposure, respectively.

							Read counts				
Target	Sample	Experiment	Timepoint	Temperature (°C)	Terbuthylazine (nM)	Replicate	Raw	Merged	Primer	Clean	Mean read length (bp)
**rbcL**	C001	Periphyton establishment	t1 (4 days)	17	-	R01	348	47	44	44	257
	C002	Periphyton establishment	t1 (4 days)	17	-	R02	11743	10814	10369	10367	263
	C003	Periphyton establishment	t1 (4 days)	17	-	R03	83641	80616	77347	77333	263
	C004	Periphyton establishment	t1 (4 days)	17	-	R04	98998	95809	91929	91907	263
	C005	Periphyton establishment	t1 (4 days)	17	-	R05	112143	108925	104458	104358	263
	C006	Periphyton establishment	t2 (18 days)	17	-	R01	74336	72056	69207	69188	263
	C007	Periphyton establishment	t2 (18 days)	17	-	R02	50325	48522	46532	46527	263
	C008	Periphyton establishment	t2 (18 days)	17	-	R03	3467	3059	2959	2958	263
	C009	Periphyton establishment	t2 (18 days)	17	-	R04	681	503	489	488	262
	C010	Periphyton establishment	t2 (18 days)	17	-	R05	60274	58377	56002	55990	263
	C011	Periphyton establishment	t3 (30 days)	17	-	R01	61525	59767	57262	57251	263
	C012	Periphyton establishment	t3 (30 days)	17	-	R02	58082	56323	54211	54201	263
	C013	Periphyton establishment	t3 (30 days)	17	-	R03	62666	61061	58652	58644	263
	C014	Periphyton establishment	t3 (30 days)	17	-	R04	78069	75921	72856	72847	263
	C015	Periphyton establishment	t3 (30 days)	17	-	R05	73409	71347	68420	68409	263
	C016	Case study	t1 (4 days)	17	0 nM	R01	3046	2766	2660	2660	263
	C017	Case study	t1 (4 days)	17	0 nM	R02	39986	38843	37251	37240	263
	C018	Case study	t1 (4 days)	17	0 nM	R03	64893	63137	60681	60671	263
	C019	Case study	t1 (4 days)	17	0 nM	R04	58465	56719	54350	54341	263
	C020	Case study	t1 (4 days)	17	1 nM	R01	35396	33967	32518	32512	263
	C021	Case study	t1 (4 days)	17	1 nM	R02	56250	54604	52538	52523	263
	C022	Case study	t1 (4 days)	17	1 nM	R03	60686	58721	56163	56149	263
	C023	Case study	t1 (4 days)	17	10 nM	R01	69620	67526	64783	64771	263
	C024	Case study	t1 (4 days)	17	10 nM	R02	90457	87712	84148	84131	263
	C025	Case study	t1 (4 days)	17	10 nM	R03	57923	56175	53649	53640	263
	C026	Case study	t1 (4 days)	17	100 nM	R01	60160	58428	56012	55999	263
	C027	Case study	t1 (4 days)	17	100 nM	R02	51991	50421	48610	48604	263
	C028	Case study	t1 (4 days)	17	100 nM	R03	49867	48016	45996	45987	263
	C029	Case study	t2 (18 days)	17	0 nM	R01	65819	63634	60939	60923	263
	C030	Case study	t2 (18 days)	17	0 nM	R02	68271	66064	63354	63343	263
	C031	Case study	t2 (18 days)	17	0 nM	R03	78131	75636	72570	72553	263
	C032	Case study	t2 (18 days)	17	0 nM	R04	57171	55448	53333	53323	263
	C033	Case study	t2 (18 days)	17	1 nM	R01	54452	52597	50476	50468	263
	C034	Case study	t2 (18 days)	17	1 nM	R02	62967	60935	58561	58547	263
	C035	Case study	t2 (18 days)	17	1 nM	R03	65104	62801	60278	60260	263

							Read counts				
Target	Sample	Experiment	Timepoint	Temperature (°C)	Terbuthylazine (nM)	Replicate	Raw	Merged	Primer	Clean	Mean read length (bp)
**rbcL**	C036	Case study	t2 (18 days)	17	10 nM	R01	52148	49857	47714	47705	263
	C037	Case study	t2 (18 days)	17	10 nM	R02	73068	70562	67609	67601	263
	C038	Case study	t2 (18 days)	17	10 nM	R03	72040	69295	66442	66427	263
	C039	Case study	t2 (18 days)	17	100 nM	R01	78897	76183	72909	72898	263
	C040	Case study	t2 (18 days)	17	100 nM	R02	82578	79966	76730	76713	263
	C041	Case study	t2 (18 days)	17	100 nM	R03	53182	51580	49558	49546	263
	C042	Case study	t3 (30 days)	17	0 nM	R01	59340	57416	55041	55028	263
	C043	Case study	t3 (30 days)	17	0 nM	R02	51778	49855	47769	47763	263
	C044	Case study	t3 (30 days)	17	0 nM	R03	55714	53201	51030	51024	263
	C045	Case study	t3 (30 days)	17	0 nM	R04	62662	60575	58048	58038	263
	C046	Case study	t3 (30 days)	17	1 nM	R01	67740	64820	62017	62005	263
	C047	Case study	t3 (30 days)	17	1 nM	R02	70501	68201	65516	65507	263
	C048	Case study	t3 (30 days)	17	1 nM	R03	66438	64061	61234	61222	263
	C049	Case study	t3 (30 days)	17	10 nM	R01	58655	56539	54249	54239	263
	C050	Case study	t3 (30 days)	17	10 nM	R02	62417	60234	57722	57709	263
	C051	Case study	t3 (30 days)	17	10 nM	R03	70655	68233	65434	65416	263
	C052	Case study	t3 (30 days)	17	100 nM	R01	64264	61765	59228	59218	263
	C053	Case study	t3 (30 days)	17	100 nM	R02	78111	75814	72799	72784	263
	C054	Case study	t3 (30 days)	17	100 nM	R03	80493	77853	74690	74671	263
	C055	Case study	t1 (4 days)	20	0 nM	R01	69391	67248	64547	64529	263
	C056	Case study	t1 (4 days)	20	0 nM	R02	84582	82166	78785	78771	263
	C057	Case study	t1 (4 days)	20	0 nM	R03	56980	55272	52951	52935	263
	C058	Case study	t1 (4 days)	20	1 nM	R01	50497	49010	46935	46925	263
	C059	Case study	t1 (4 days)	20	1 nM	R02	44083	42511	40849	40844	263
	C060	Case study	t1 (4 days)	20	1 nM	R03	48906	47119	45223	45211	263
	C061	Case study	t1 (4 days)	20	10 nM	R01	50801	49270	47158	47146	263
	C062	Case study	t1 (4 days)	20	10 nM	R02	48894	47210	45144	45130	263
	C063	Case study	t1 (4 days)	20	10 nM	R03	55174	53509	50956	50948	263
	C064	Case study	t1 (4 days)	20	100 nM	R01	66739	64607	61674	61662	263
	C065	Case study	t1 (4 days)	20	100 nM	R02	72113	69356	66344	66331	263
	C066	Case study	t1 (4 days)	20	100 nM	R03	66981	64568	61851	61841	263
	C067	Case study	t2 (18 days)	20	0 nM	R01	62344	59817	57328	57315	263
	C068	Case study	t2 (18 days)	20	0 nM	R02	72479	69380	66706	66689	263
	C069	Case study	t2 (18 days)	20	0 nM	R03	82242	79188	75880	75865	263
	C070	Case study	t2 (18 days)	20	1 nM	R01	74035	71122	68181	68166	263
	C071	Case study	t2 (18 days)	20	1 nM	R02	82343	79257	76034	76022	263
	C072	Case study	t2 (18 days)	20	1 nM	R03	82936	80099	76837	76823	263
	C073	Case study	t2 (18 days)	20	10 nM	R01	49536	47386	45049	45040	263
	C074	Case study	t2 (18 days)	20	10 nM	R02	75976	72787	69115	69096	263
	C075	Case study	t2 (18 days)	20	10 nM	R03	68246	65282	62055	62036	263
	C076	Case study	t2 (18 days)	20	100 nM	R01	66976	63843	60862	60854	263

							Read counts				
Target	Sample	Experiment	Timepoint	Temperature (°C)	Terbuthylazine (nM)	Replicate	Raw	Merged	Primer	Clean	Mean read length (bp)
**rbcL**	C077	Case study	t2 (18 days)	20	100 nM	R02	75584	72540	69065	69046	263
	C078	Case study	t2 (18 days)	20	100 nM	R03	76164	73000	69725	69702	263
	C079	Case study	t3 (30 days)	20	0 nM	R01	73411	70484	67101	67088	263
	C080	Case study	t3 (30 days)	20	0 nM	R02	72586	69655	66444	66431	263
	C081	Case study	t3 (30 days)	20	0 nM	R03	56519	54345	51778	51772	263
	C082	Case study	t3 (30 days)	20	1 nM	R01	71381	68512	65287	65263	263
	C083	Case study	t3 (30 days)	20	1 nM	R02	71424	68565	65315	65304	263
	C084	Case study	t3 (30 days)	20	1 nM	R03	79745	76197	72545	72535	263
	C085	Case study	t3 (30 days)	20	10 nM	R01	85023	81748	77966	77950	263
	C086	Case study	t3 (30 days)	20	10 nM	R02	86112	82599	78789	78777	263
	C087	Case study	t3 (30 days)	20	10 nM	R03	84654	81397	77632	77619	263
	C088	Case study	t3 (30 days)	20	100 nM	R01	50025	48097	45894	45879	263
	C089	Case study	t3 (30 days)	20	100 nM	R02	417	168	163	163	261
	C090	Case study	t3 (30 days)	20	100 nM	R03	1817	1475	1399	1399	263
	NegPCR1a	negative control					1042	683	641	641	263
	Pos-mock4	mock4 community					391384	377320	361632	361552	263
	Pos-pool-cyano	mock community of 4 cyanobacteria species					588	290	277	277	262
	Pos-pool-chloro	mock community of 6 green algae species					304340	293153	280559	280505	263
	Pos-pool-diatom	mock community of 16 diatom species					82871	80186	76854	76846	263
	Pos-pool-all	mock community of all species					70628	67927	65127	65110	263

Supplementary Table 11. Read processing reports for 16S rRNA gene. Read counts for each sample for each of the processing steps. The mean length of the reads is also indicated in the table. The composition of mock communities used as positive controls is described in SI Table 5. 0 nM, 1 nM, 10 nM and 100 nM correspond to 0 nM (unexposed control), 1 nM, 10 nM and 100 nM terbuthylezine exposure, respectively.

							Read counts				
Target	Sample	Experiment	Timepoint	Temperature (°C)	Terbuthylazine (nM)	Replicate	Raw	Merged	Primer	Clean	Mean read length (bp)
**16S rRNA gene**	C001	Periphyton establishment	t1 (4 days)	17	-	R01	73948	70628	69617	69569	386
	C002	Periphyton establishment	t1 (4 days)	17	-	R02	68998	66687	65633	65590	387
	C003	Periphyton establishment	t1 (4 days)	17	-	R03	68606	66061	65003	64963	388
	C004	Periphyton establishment	t1 (4 days)	17	-	R04	59659	57255	56334	56287	389
	C005	Periphyton establishment	t1 (4 days)	17	-	R05	77810	75426	74259	74202	387
	C006	Periphyton establishment	t2 (18 days)	17	-	R01	80725	77734	76370	76323	381
	C007	Periphyton establishment	t2 (18 days)	17	-	R02	67215	64727	63632	63590	381
	C008	Periphyton establishment	t2 (18 days)	17	-	R03	77042	74305	73160	73122	381
	C009	Periphyton establishment	t2 (18 days)	17	-	R04	19150	18160	17844	17832	380
	C010	Periphyton establishment	t2 (18 days)	17	-	R05	67964	64176	62999	62967	381
	C011	Periphyton establishment	t3 (30 days)	17	-	R01	66035	63410	62175	62140	380
	C012	Periphyton establishment	t3 (30 days)	17	-	R02	59673	56793	55854	55819	380
	C013	Periphyton establishment	t3 (30 days)	17	-	R03	66850	64281	63117	63083	380
	C014	Periphyton establishment	t3 (30 days)	17	-	R04	65839	63216	62092	62051	380
	C015	Periphyton establishment	t3 (30 days)	17	-	R05	62854	60296	59261	59222	380
	C016	Case study	t1 (4 days)	17	0 nM	R01	65242	63216	62187	62142	381
	C017	Case study	t1 (4 days)	17	0 nM	R02	82744	80361	79117	79071	382
	C018	Case study	t1 (4 days)	17	0 nM	R03	71993	69965	68824	68788	381
	C019	Case study	t1 (4 days)	17	0 nM	R04	61792	59930	58996	58951	381
	C020	Case study	t1 (4 days)	17	1 nM	R01	43414	42000	41316	41286	382
	C021	Case study	t1 (4 days)	17	1 nM	R02	65013	63215	62194	62164	382
	C022	Case study	t1 (4 days)	17	1 nM	R03	58885	57136	56061	56025	382
	C023	Case study	t1 (4 days)	17	10 nM	R01	65390	63477	62459	62415	382
	C024	Case study	t1 (4 days)	17	10 nM	R02	67679	65403	64305	64266	382
	C025	Case study	t1 (4 days)	17	10 nM	R03	119668	115764	113802	113715	382
	C026	Case study	t1 (4 days)	17	100 nM	R01	68398	66505	65434	65389	382
	C027	Case study	t1 (4 days)	17	100 nM	R02	70337	68329	67246	67205	382
	C028	Case study	t1 (4 days)	17	100 nM	R03	54721	52934	52120	52079	382
	C029	Case study	t2 (18 days)	17	0 nM	R01	71510	69128	67881	67841	381
	C030	Case study	t2 (18 days)	17	0 nM	R02	72444	69847	68560	68520	381
	C031	Case study	t2 (18 days)	17	0 nM	R03	70867	68310	67087	67040	381
	C032	Case study	t2 (18 days)	17	0 nM	R04	77765	75372	74164	74116	381
	C033	Case study	t2 (18 days)	17	1 nM	R01	74753	71729	70573	70527	380
	C034	Case study	t2 (18 days)	17	1 nM	R02	64276	61809	60737	60694	380
	C035	Case study	t2 (18 days)	17	1 nM	R03	65567	62830	61654	61618	380
	C036	Case study	t2 (18 days)	17	10 nM	R01	53490	51156	50238	50212	381

Target	Sample	Experiment	Timepoint	Temperature (°C)	Terbuthylazine (nM)	Replicate	Read counts				Mean read length (bp)
							Raw	Merged	Primer	Clean	
**16S rRNA gene**	C037	Case study	t2 (18 days)	17	10 nM	R02	70987	68540	67287	67241	381
	C038	Case study	t2 (18 days)	17	10 nM	R03	72039	69474	68192	68154	381
	C039	Case study	t2 (18 days)	17	100 nM	R01	71533	69320	68121	68078	382
	C040	Case study	t2 (18 days)	17	100 nM	R02	69818	67674	66554	66517	382
	C041	Case study	t2 (18 days)	17	100 nM	R03	81306	78464	77191	77142	382
	C042	Case study	t3 (30 days)	17	0 nM	R01	65790	63036	61861	61824	381
	C043	Case study	t3 (30 days)	17	0 nM	R02	68312	64982	63685	63649	380
	C044	Case study	t3 (30 days)	17	0 nM	R03	60859	57258	56110	56082	380
	C045	Case study	t3 (30 days)	17	0 nM	R04	66208	63342	62209	62164	380
	C046	Case study	t3 (30 days)	17	1 nM	R01	61002	57832	56729	56694	380
	C047	Case study	t3 (30 days)	17	1 nM	R02	60992	58317	57310	57270	381
	C048	Case study	t3 (30 days)	17	1 nM	R03	59019	55943	54921	54886	380
	C049	Case study	t3 (30 days)	17	10 nM	R01	79697	75831	74603	74558	380
	C050	Case study	t3 (30 days)	17	10 nM	R02	71619	68592	67292	67254	380
	C051	Case study	t3 (30 days)	17	10 nM	R03	75861	72643	71212	71160	380
	C052	Case study	t3 (30 days)	17	100 nM	R01	66094	63633	62583	62537	381
	C053	Case study	t3 (30 days)	17	100 nM	R02	78682	75900	74497	74443	381
	C054	Case study	t3 (30 days)	17	100 nM	R03	78070	75367	74140	74084	381
	C055	Case study	t1 (4 days)	20	0 nM	R01	63893	62099	61113	61075	382
	C056	Case study	t1 (4 days)	20	0 nM	R02	75580	73481	72364	72301	382
	C057	Case study	t1 (4 days)	20	0 nM	R03	67864	66005	65016	64966	382
	C058	Case study	t1 (4 days)	20	1 nM	R01	53268	51806	50997	50962	382
	C059	Case study	t1 (4 days)	20	1 nM	R02	50648	49187	48359	48312	382
	C060	Case study	t1 (4 days)	20	1 nM	R03	47914	46366	45629	45602	382
	C061	Case study	t1 (4 days)	20	10 nM	R01	47085	45750	45018	44985	382
	C062	Case study	t1 (4 days)	20	10 nM	R02	47414	45995	45232	45204	382
	C063	Case study	t1 (4 days)	20	10 nM	R03	47319	45880	45197	45164	382
	C064	Case study	t1 (4 days)	20	100 nM	R01	53556	51820	43016	42988	382
	C065	Case study	t1 (4 days)	20	100 nM	R02	80388	77480	61109	61067	382
	C066	Case study	t1 (4 days)	20	100 nM	R03	73634	71260	67842	67798	382
	C067	Case study	t2 (18 days)	20	0 nM	R01	78380	75374	74085	74029	381
	C068	Case study	t2 (18 days)	20	0 nM	R02	80018	76551	75279	75227	381
	C069	Case study	t2 (18 days)	20	0 nM	R03	89186	85670	84227	84172	381
	C070	Case study	t2 (18 days)	20	1 nM	R01	74458	71568	70392	70353	381
	C071	Case study	t2 (18 days)	20	1 nM	R02	74194	71373	70203	70154	381
	C072	Case study	t2 (18 days)	20	1 nM	R03	66191	63430	62411	62365	381
	C073	Case study	t2 (18 days)	20	10 nM	R01	91425	88314	86951	86887	381
	C074	Case study	t2 (18 days)	20	10 nM	R02	85144	82410	81070	81015	381
	C075	Case study	t2 (18 days)	20	10 nM	R03	90017	87043	85597	85541	381
	C076	Case study	t2 (18 days)	20	100 nM	R01	70790	68275	67226	67190	382
	C077	Case study	t2 (18 days)	20	100 nM	R02	87120	84655	83269	83217	382
	C078	Case study	t2 (18 days)	20	100 nM	R03	77825	75531	74389	74336	382

Target	Sample	Experiment	Timepoint	Temperature (°C)	Terbuthylazine (nM)	Replicate	Read counts Raw	Merged	Primer	Clean	Mean read length (bp)
**16S rRNA gene**	C079	Case study	t3 (30 days)	20	0 nM	R01	74161	71202	69941	69900	381
	C080	Case study	t3 (30 days)	20	0 nM	R02	72932	70145	68981	68932	381
	C081	Case study	t3 (30 days)	20	0 nM	R03	87063	83601	82294	82240	381
	C082	Case study	t3 (30 days)	20	1 nM	R01	80364	77482	76123	76068	381
	C083	Case study	t3 (30 days)	20	1 nM	R02	79837	76646	75218	75163	381
	C084	Case study	t3 (30 days)	20	1 nM	R03	71350	68269	67114	67070	381
	C085	Case study	t3 (30 days)	20	10 nM	R01	79870	76914	75569	75524	381
	C086	Case study	t3 (30 days)	20	10 nM	R02	76825	73970	72677	72624	381
	C087	Case study	t3 (30 days)	20	10 nM	R03	73757	71039	69889	69853	381
	C088	Case study	t3 (30 days)	20	100 nM	R01	70245	67784	66640	66595	381
	C089	Case study	t3 (30 days)	20	100 nM	R02	16447	15561	15268	15257	381
	C090	Case study	t3 (30 days)	20	100 nM	R03	84753	81949	80627	80571	381
	NegPCR1a	negative control					64394	62261	61286	61240	382
	Pos-mock4	mock4 community					62579	58772	57636	57599	398
	Pos-pool-cyano	mock community of 4 cyanobacteria species					76863	73888	72665	72618	380
	Pos-pool-chloro	mock community of 6 green algae species					89884	84753	83561	83513	379
	Pos-pool-diatom	mock community of 16 diatom species					96290	92264	90754	90696	378
	Pos-pool-all	mock community of all species					74204	71831	70721	70683	381

Supplementary Table 12. Two-way ANOVA results of the single species relative abundances (n = 3) during periphyton formation (t1: 4 days, t2: 18 days and t3: 30 days) upon exposure to the herbicide terbuthylzine (Herbicide), increased temperature from 17 °C to 20 °C (Temprature) and their combination (Herbicide x Temperature). ns: not significant.

Group	Species name	Time (t)	Herbicide	Temperature	Hebicide x Temperature
A	*Achnanthidium pyrenaicum*	t1	F (3, 16) = 43.49, P<0.0001	F (1, 16) = 25.44, P=0.001	F (3, 16) = 4.59, P=0.017
		t2	F (3, 16) = 21.00, P<0.0001	F (1, 16) = 0.01, ns	F (3, 16) = 1.44, ns
		t3	F (3, 16) = 4.16, P=0.023	F (1, 16) = 0.56, ns	F (3, 16) = 1.85, ns
	Cyclotella meneghiniana	t1	F (3, 16) = 38.74, P<0.0001	F (1, 16) = 40.51, P<0.0001	F (3, 16) = 13.81, P=0.001
		t2	F (3, 16) = 78.73, P<0.0001	F (1, 16) = 57.77, P<0.0001	F (3, 16) = 59.47, P<0.0001
		t3	F (3, 16) = 2.68, ns	F (1, 16) = 16.01, P=0.001	F (3, 16) = 1.89, ns
B	*Ulnaria ulna*	t2	F (3, 16) = 50.58, P<0.0001	F (1, 16) = 29.03, P<0.0001	F (3, 16) = 10.48, P=0.005
		t3	F (3, 16) = 0.76, ns	F (1, 16) = 3.82, ns	F (3, 16) = 0.21, ns
	Tabellaria sp.	t2	F (3, 16) = 16.18, P<0.0001	F (1, 16) = 1.71, ns	F (3, 16) = 5.54, P=0.008
		t3	F (3, 16) = 1.65, ns	F (1, 16) = 125.90, P<0.0001	F (3, 16) = 1.65, ns
	Synedra sp.	t2	F (3, 16) = 25.80, P<0.0001	F (1, 16) = 26.00, P=0.001	F (3, 16) = 15.31, P<0.0001
		t3	F (3, 16) = 0.09, ns	F (1, 16) = 1.85, ns	F (3, 16) = 6.14, P=0.005
	Melosira sp.	t2	F (3, 16) = 33.86, P<0.0001	F (1, 16) = 2.33, ns	F (3, 16) = 2.18, ns
		t3	F (3, 16) = 2.34, ns	F (1, 16) = 79.98, P<0.0001	F (3, 16) = 1.51, ns
	Cymbella cistula	t2	F (3, 16) = 41.68, P<0.0001	F (1, 16) = 1.05, ns	F (3, 16) = 8.11, P=0.001
		t3	F (3, 16) = 0.62, ns	F (1, 16) = 5.74, P=0.029	F (3, 16) = 1.01, ns
	Fragilaria sp.	t2	F (3, 16) = 14.96, P<0.0001	F (1, 16) = 5.30, P=0.035	F (3, 16) = 5.55, P=0.008
		t3	F (3, 16) = 5.16, P=0.011	F (1, 16) = 1.85, ns	F (3, 16) = 1.27, ns
	Gomphonema parvulum	t2	F (3, 16) = 64.59, P<0.0001	F (1, 16) = 15.18, P=0.001	F (3, 16) = 19.85, P<0.0001
		t3	F (3, 16) = 6.58, P=0.0042	F (1, 16) = 12.87, P=0.002	F (3, 16) = 5.10, P=0.011
	Gomphonema clavatum	t2	F (3, 16) = 111.60, P<0.0001	F (1, 16) = 53.73, P<0.0001	F (3, 16) = 24.55, P<0.0001
		t3	F (3, 16) = 1.53, ns	F (1, 16) = 26.00, P=0.001	F (3, 16) = 2.54, ns
C	*Botryococcus braunii*	t3	F (3, 16) = 53.34, P<0.0001	F (1, 16) = 13.86, P=0.002	F (3, 16) = 3.77, P=0.032
	Chlorella vulgaris	t3	F (3, 16) = 5.56, P=0.008	F (1, 16) = 64.08, P<0.0001	F (3, 16) = 5.60, P=0.008
	Scenedesmus vacuolatus	t3	F (3, 16) = 16.15, P<0.0001	F (1, 16) = 74.69, P<0.0001	F (3, 16) = 3.27, P=0.048
	Scenedesmus acuminatus	t3	F (3, 16) = 8.47, P=0.001	F (1, 16) = 96.34, P<0.0001	F (3, 16) = 4.26, P=0.021
	Pediastrum sp.	t3	F (3, 16) = 7.34, P=0.003	F (1, 16) = 18.14, P=0.0006	F (3, 16) = 2.93, ns
	Sellaphora minima	t3	F (3, 16) = 3.83, P=0.030	F (1, 16) = 4.37, P=0.052	F (3, 16) = 2.87, ns
	Craticula cuspidata	t3	F (3, 16) = 0.34, ns	F (1, 16) = 9.39, P=0.007	F (3, 16) = 0.85, ns
	Craticula accomoda	t3	F (3, 16) = 3.89, P=0.029	F (1, 16) = 12.70, P=0.002	F (3, 16) = 1.83, ns
	Nitzschia vermicularis	t3	F (3, 16) = 2.59, ns	F (1, 16) = 61.73, P<0.0001	F (3, 16) = 0.67, ns
	Nitzschia palea	t3	F (3, 16) = 2.16, ns	F (1, 16) = 8.627, P=0.001	F (3, 16) = 2.60, ns
	Chamaesiphon polonicus	t3	F (3, 16) = 41.31, P<0.0001	F (1, 16) = 31.82, P<0.0001	F (3, 16) = 0.47, ns
	Merismopedia glauca	t3	F (3, 16) = 0.18, ns	F (1, 16) = 2.54, ns	F (3, 16) = 2.11, ns
	Pseudanabaena galeata	t3	F (3, 16) = 0.48, ns	F (1, 16) = 5.81, P=0.0283	F (3, 16) = 1.83, ns
	Phormidium sp.	t3	F (3, 16) = 12.10, P=0.0002	F (1, 16) = 81.06, P<0.0001	F (3, 16) = 9.86, P=0.0006

Supplementary Table 13. Tukey's pairwise comparison at sampling time t1 (4 days of periphyton establishment) following the changes in species relative abundances (n = 3) upon exposure to the herbicide terbuthylzine (0, 1, 10 or 100 nM), at 17 °C or 20 °C. ns: not significant.

Group A	Species name			
	A. pyrenaicum		*C. meneghiniana*	
	P value		P value	
17 °C:0 nM vs. 17 °C:1 nM	<0.0001	****	<0.0001	****
17 °C:0 nM vs. 17 °C:10 nM	<0.0001	****	<0.0001	****
17 °C:0 nM vs. 17 °C:100 nM	0.0003	***	0.006	**
17 °C:0 nM vs. 20 °C:0 nM	<0.0001	****	<0.0001	****
17 °C:0 nM vs. 20 °C:1 nM	<0.0001	****	<0.0001	****
17 °C:0 nM vs. 20 °C:10 nM	<0.0001	****	<0.0001	****
17 °C:0 nM vs. 20 °C:100 nM	<0.0001	****	0.0005	***
17 °C:1 nM vs. 17 °C:10 nM	0.0499	*	0.0528	ns
17 °C:1 nM vs. 17 °C:100 nM	0.0003	***	0.0003	***
17 °C:1 nM vs. 20 °C:0 nM	0.4289	ns	0.4588	ns
17 °C:1 nM vs. 20 °C:1 nM	0.9461	ns	0.9314	ns
17 °C:1 nM vs. 20 °C:10 nM	0.6439	ns	0.6458	ns
17 °C:1 nM vs. 20 °C:100 nM	0.0028	**	0.0032	**
17 °C:10 nM vs. 17 °C:100 nM	0.1718	ns	0.1862	ns
17 °C:10 nM vs. 20 °C:0 nM	0.8795	ns	0.8695	ns
17 °C:10 nM vs. 20 °C:1 nM	0.0059	**	0.0056	**
17 °C:10 nM vs. 20 °C:10 nM	0.6941	ns	0.7093	ns
17 °C:10 nM vs. 20 °C:100 nM	0.7991	ns	0.825	ns
17 °C:100 nM vs. 20 °C:0 nM	0.0152	*	0.0159	*
17 °C:100 nM vs. 20 °C:1 nM	<0.0001	****	<0.0001	****
17 °C:100 nM vs. 20 °C:10 nM	0.0074	**	0.0085	**
17 °C:100 nM vs. 20 °C:100 nM	0.8965	ns	0.8952	ns
20 °C:0 nM vs. 20 °C:1 nM	0.0737	ns	0.0734	ns
20 °C:0 nM vs. 20 °C:10 nM	>0.9999	ns	>0.9999	ns
20 °C:0 nM vs. 20 °C:100 nM	0.1593	ns	0.167	ns
20 °C:1 nM vs. 20 °C:10 nM	0.1421	ns	0.1297	ns
20 °C:1 nM vs. 20 °C:100 nM	0.0003	***	0.0004	***
20 °C:10 nM vs. 20 °C:100 nM	0.0834	ns	0.0962	ns

Supplementary Table 14. Tukey's pairwise comparison at sampling time t2 (18 days of periphyton establishment) following the changes in species relative abundances (n = 3) upon exposure to the herbicide terbuthylzine (0, 1, 10 or 100 nM), at 17 °C or 20 °C. ns: not significant.

**Group A**	**Species name**	
	A. pyrenaicum	***C. meneghiniana***
	P value	P value
17 °C:0 nM vs. 17 °C:1 nM	0.11 ns	<0.0001 ****
17 °C:0 nM vs. 17 °C:10 nM	0.3212 ns	0.3628 ns
17 °C:0 nM vs. 17 °C:100 nM	0.9993 ns	0.3063 ns
17 °C:0 nM vs. 20 °C:0 nM	0.5384 ns	>0.9999 ns
17 °C:0 nM vs. 20 °C:1 nM	0.1161 ns	0.1617 ns
17 °C:0 nM vs. 20 °C:10 nM	0.513 ns	0.5725 ns
17 °C:0 nM vs. 20 °C:100 nM	0.9968 ns	0.0954 ns
17 °C:1 nM vs. 17 °C:10 nM	0.9968 ns	<0.0001 ****
17 °C:1 nM vs. 17 °C:100 nM	0.2601 ns	<0.0001 ****
17 °C:1 nM vs. 20 °C:0 nM	0.953 ns	<0.0001 ****
17 °C:1 nM vs. 20 °C:1 nM	>0.9999 ns	<0.0001 ****
17 °C:1 nM vs. 20 °C:10 nM	0.9619 ns	<0.0001 ****
17 °C:1 nM vs. 20 °C:100 nM	0.3224 ns	<0.0001 ****
17 °C:10 nM vs. 17 °C:100 nM	0.6059 ns	>0.9999 ns
17 °C:10 nM vs. 20 °C:0 nM	0.9999 ns	0.3105 ns
17 °C:10 nM vs. 20 °C:1 nM	0.9976 ns	0.9993 ns
17 °C:10 nM vs. 20 °C:10 nM	>0.9999 ns	>0.9999 ns
17 °C:10 nM vs. 20 °C:100 nM	0.6923 ns	0.988 ns
17 °C:100 nM vs. 20 °C:0 nM	0.8314 ns	0.2598 ns
17 °C:100 nM vs. 20 °C:1 nM	0.2726 ns	0.9999 ns
17 °C:100 nM vs. 20 °C:10 nM	0.8106 ns	0.9995 ns
17 °C:100 nM vs. 20 °C:100 nM	>0.9999 ns	0.9952 ns
20 °C:0 nM vs. 20 °C:1 nM	0.9595 ns	0.1339 ns
20 °C:0 nM vs. 20 °C:10 nM	>0.9999 ns	0.5071 ns
20 °C:0 nM vs. 20 °C:100 nM	0.8927 ns	0.0781 ns
20 °C:1 nM vs. 20 °C:10 nM	0.9675 ns	0.9807 ns
20 °C:1 nM vs. 20 °C:100 nM	0.3369 ns	>0.9999 ns
20 °C:10 nM vs. 20 °C:100 nM	0.876 ns	0.916 ns

**Group B**	**Species name**							
	G. parvulum	***G. clavatum***	***Synedra sp.***	***Fragilaria sp.***	***Melosira sp.***	***C. cistula***	***Tabellaria sp.***	***U. ulna***
	P value	P value	P value	P value	P value	P value	P value	P value
17 °C:0 nM vs. 17 °C:1 nM	<0.0001 ****	<0.0001 ****	<0.0001 ****	<0.0001 ****	0.0005 ***	<0.0001 ****	<0.0001 ****	<0.0001 ****
17 °C:0 nM vs. 17 °C:10 nM	0.7814 ns	0.4238 ns	0.9458 ns	0.9821 ns	0.7658 ns	0.0358 *	0.9997 ns	0.0286 *
17 °C:0 nM vs. 17 °C:100 nM	>0.9999 ns	0.6409 ns	>0.9999 ns	0.9995 ns	0.0011 **	>0.9999 ns	>0.9999 ns	>0.9999 ns
17 °C:0 nM vs. 20 °C:0 nM	0.9391 ns	>0.9999 ns	0.2897 ns	0.8072 ns	0.7658 ns	>0.9999 ns	>0.9999 ns	0.8085 ns

Group B	Species name															
	G. parvulum		*G. clavatum*		*Synedra sp.*		*Fragilaria sp.*		*Melosira sp.*		*C. cistula*		*Tabellaria sp.*		*U. ulna*	
	P value		P value		P value		P value		P value		P value		P value		P value	
17 °C:0 nM vs. 20 °C:1 nM	0.029	*	0.1268	ns	>0.9999	ns	0.4308	ns	>0.9999	ns	0.0033	**	0.0659	ns	0.0849	ns
17 °C:0 nM vs. 20 °C:10 nM	0.7403	ns	0.9147	ns	>0.9999	ns	0.8201	ns	>0.9999	ns	0.6977	ns	0.191	ns	0.5805	ns
17 °C:0 nM vs. 20 °C:100 nM	0.6759	ns	0.9861	ns	0.198	ns	0.9913	ns	0.0008	***	0.8613	ns	0.4109	ns	0.9866	ns
17 °C:1 nM vs. 17 °C:10 nM	<0.0001	****	<0.0001	****	<0.0001	****	<0.0001	****	<0.0001	****	<0.0001	****	<0.0001	****	<0.0001	****
17 °C:1 nM vs. 17 °C:100 nM	<0.0001	****	<0.0001	****	<0.0001	****	<0.0001	****	<0.0001	****	<0.0001	****	<0.0001	****	<0.0001	****
17 °C:1 nM vs. 20 °C:0 nM	<0.0001	****	<0.0001	****	<0.0001	****	<0.0001	****	<0.0001	****	<0.0001	****	<0.0001	****	<0.0001	****
17 °C:1 nM vs. 20 °C:1 nM	<0.0001	****	<0.0001	****	<0.0001	****	<0.0001	****	0.0003	***	<0.0001	****	0.005	**	<0.0001	****
17 °C:1 nM vs. 20 °C:10 nM	<0.0001	****	<0.0001	****	<0.0001	****	<0.0001	****	0.0004	***	<0.0001	****	0.0016	**	<0.0001	****
17 °C:1 nM vs. 20 °C:100 nM	<0.0001	****	<0.0001	****	<0.0001	****	<0.0001	****	<0.0001	****	<0.0001	****	0.0006	***	<0.0001	****
17 °C:10 nM vs. 17 °C:100 nM	0.7638	ns	>0.9999	ns	0.9884	ns	0.8475	ns	0.0217	*	0.0426	*	>0.9999	ns	0.0587	ns
17 °C:10 nM vs. 20 °C:0 nM	0.203	ns	0.521	ns	0.0431	*	0.3146	ns	>0.9999	ns	0.074	ns	>0.9999	ns	0.0016	**
17 °C:10 nM vs. 20 °C:1 nM	0.3904	ns	0.9912	ns	0.9945	ns	0.9057	ns	0.8697	ns	0.909	ns	0.1502	ns	0.9987	ns
17 °C:10 nM vs. 20 °C:10 nM	>0.9999	ns	0.9773	ns	0.9627	ns	0.999	ns	0.8566	ns	0.5357	ns	0.3809	ns	0.5841	ns
17 °C:10 nM vs. 20 °C:100 nM	>0.9999	ns	0.8869	ns	0.759	ns	>0.9999	ns	0.017	*	0.3611	ns	0.679	ns	0.1364	ns
17 °C:100 nM vs. 20 °C:0 nM	0.9474	ns	0.7406	ns	0.1887	ns	0.9707	ns	0.0217	*	>0.9999	ns	>0.9999	ns	0.595	ns
17 °C:100 nM vs. 20 °C:1 nM	0.0272	*	0.9301	ns	>0.9999	ns	0.2114	ns	0.0016	**	0.0039	**	0.0954	ns	0.1643	ns
17 °C:100 nM vs. 20 °C:10 nM	0.7217	ns	0.9989	ns	>0.9999	ns	0.5398	ns	0.0015	**	0.7491	ns	0.2626	ns	0.7963	ns
17 °C:100 nM vs. 20 °C:100 nM	0.6564	ns	0.9792	ns	0.3025	ns	0.8909	ns	>0.9999	ns	0.8971	ns	0.5245	ns	0.9997	ns
20 °C:0 nM vs. 20 °C:1 nM	0.0032	**	0.17	ns	0.1604	ns	0.037	*	0.8697	ns	0.0069	**	0.0954	ns	0.005	**
20 °C:0 nM vs. 20 °C:10 nM	0.1793	ns	0.9595	ns	0.2575	ns	0.1301	ns	0.8566	ns	0.8887	ns	0.2626	ns	0.0607	ns
20 °C:0 nM vs. 20 °C:100 nM	0.1486	ns	0.9963	ns	0.0021	**	0.3649	ns	0.017	*	0.9722	ns	0.5245	ns	0.3365	ns
20 °C:1 nM vs. 20 °C:10 nM	0.4307	ns	0.6708	ns	>0.9999	ns	0.9961	ns	>0.9999	ns	0.0821	ns	0.9982	ns	0.8888	ns
20 °C:1 nM vs. 20 °C:100 nM	0.4937	ns	0.4597	ns	0.3479	ns	0.8653	ns	0.0013	**	0.0454	*	0.9394	ns	0.3394	ns
20 °C:10 nM vs. 20 °C:100 nM	>0.9999	ns	>0.9999	ns	0.2245	ns	0.997	ns	0.0012	**	>0.9999	ns	0.9993	ns	0.9625	ns

Supplementary Table 15. Tukey's pairwise comparison at sampling time t3 (30 days of periphyton establishment) following the changes in the species relative abundances (n = 3) upon exposure to the herbicide terbuthylzine (0, 1, 10 or 100 nM), at 17 °C or 20 °C. ns: not significant.

Group A	Species name			
	A. pyrenaicum		*C. meneghiniana*	
	P value		P value	
17 °C:0 nM vs. 17 °C:1 nM	0.1899	ns	0.9998	ns
17 °C:0 nM vs. 17 °C:10 nM	0.2029	ns	0.6774	ns
17 °C:0 nM vs. 17 °C:100 nM	0.2074	ns	0.0889	ns
17 °C:0 nM vs. 20 °C:0 nM	0.4714	ns	0.0622	ns
17 °C:0 nM vs. 20 °C:1 nM	0.1775	ns	0.1995	ns
17 °C:0 nM vs. 20 °C:10 nM	0.2345	ns	0.2377	ns
17 °C:0 nM vs. 20 °C:100 nM	0.9792	ns	0.0439	*
17 °C:1 nM vs. 17 °C:10 nM	>0.9999	ns	0.8972	ns
17 °C:1 nM vs. 17 °C:100 nM	>0.9999	ns	0.0389	*
17 °C:1 nM vs. 20 °C:0 nM	0.9976	ns	0.0267	*
17 °C:1 nM vs. 20 °C:1 nM	>0.9999	ns	0.0931	ns
17 °C:1 nM vs. 20 °C:10 nM	>0.9999	ns	0.1134	ns
17 °C:1 nM vs. 20 °C:100 nM	0.638	ns	0.0186	*
17 °C:10 nM vs. 17 °C:100 nM	>0.9999	ns	0.0033	**
17 °C:10 nM vs. 20 °C:0 nM	0.9984	ns	0.0023	**
17 °C:10 nM vs. 20 °C:1 nM	>0.9999	ns	0.0083	**
17 °C:10 nM vs. 20 °C:10 nM	>0.9999	ns	0.0103	*
17 °C:10 nM vs. 20 °C:100 nM	0.6624	ns	0.0016	**
17 °C:100 nM vs. 20 °C:0 nM	0.9986	ns	>0.9999	ns
17 °C:100 nM vs. 20 °C:1 nM	>0.9999	ns	0.9996	ns
17 °C:100 nM vs. 20 °C:10 nM	>0.9999	ns	0.9986	ns
17 °C:100 nM vs. 20 °C:100 nM	0.6704	ns	>0.9999	ns
20 °C:0 nM vs. 20 °C:1 nM	0.9965	ns	0.9968	ns
20 °C:0 nM vs. 20 °C:10 nM	0.9995	ns	0.9919	ns
20 °C:0 nM vs. 20 °C:100 nM	0.9359	ns	>0.9999	ns
20 °C:1 nM vs. 20 °C:10 nM	>0.9999	ns	>0.9999	ns
20 °C:1 nM vs. 20 °C:100 nM	0.6135	ns	0.9862	ns
20 °C:10 nM vs. 20 °C:100 nM	0.7154	ns	0.973	ns

Group B	Species name															
	G. parvulum		*G. clavatum*		*Synedra sp.*		*Fragilaria sp.*		*Melosira sp.*		*C. cistula*		*Tabellaria sp.*		*U. ulna*	
	P value		P value		P value		P value		P value		P value		P value		P value	
17 °C:0 nM vs. 17 °C:1 nM	0.8139	ns	0.4908	ns	>0.9999	ns	>0.9999	ns	>0.9999	ns	0.6528	ns	>0.9999	ns	>0.9999	ns
17 °C:0 nM vs. 17 °C:10 nM	0.8945	ns	0.8623	ns	>0.9999	ns	0.9643	ns	>0.9999	ns	>0.9999	ns	>0.9999	ns	0.9647	ns
17 °C:0 nM vs. 17 °C:100 nM	0.7807	ns	0.9738	ns	>0.9999	ns	0.8178	ns	>0.9999	ns	>0.9999	ns	>0.9999	ns	>0.9999	ns

Group B	Species name															
	G. parvulum		*G. clavatum*		*Synedra sp.*		*Fragilaria sp.*		*Melosira sp.*		*C. cistula*		*Tabellaria sp.*		*U. ulna*	
	P value		P value		P value		P value		P value		P value		P value		P value	
17 °C:0 nM vs. 20 °C:0 nM	0.9931	ns	0.8543	ns	>0.9999	ns	>0.9999	ns	0.0076	**	0.7414	ns	0.9989	ns	>0.9999	ns
17 °C:0 nM vs. 20 °C:1 nM	0.6306	ns	0.8953	ns	>0.9999	ns	0.6317	ns	0.004	**	0.9786	ns	0.781	ns	0.9767	ns
17 °C:0 nM vs. 20 °C:10 nM	0.4412	ns	0.9508	ns	0.5688	ns	0.9805	ns	0.0008	***	0.0341	*	0.0777	ns	0.982	ns
17 °C:0 nM vs. 20 °C:100 nM	0.7515	ns	0.9626	ns	0.9754	ns	0.9994	ns	0.4181	ns	0.3142	ns	0.0427	*	0.2598	ns
17 °C:1 nM vs. 17 °C:10 nM	>0.9999	ns	0.9966	ns	>0.9999	ns	0.9969	ns	>0.9999	ns	0.551	ns	>0.9999	ns	0.9854	ns
17 °C:1 nM vs. 17 °C:100 nM	0.1162	ns	0.1164	ns	>0.9999	ns	0.6204	ns	>0.9999	ns	0.4431	ns	>0.9999	ns	>0.9999	ns
17 °C:1 nM vs. 20 °C:0 nM	0.9956	ns	0.0548	ns	>0.9999	ns	0.9947	ns	0.0143	*	>0.9999	ns	0.9989	ns	>0.9999	ns
17 °C:1 nM vs. 20 °C:1 nM	>0.9999	ns	0.0663	ns	>0.9999	ns	0.8269	ns	0.0076	**	0.9882	ns	0.781	ns	0.9914	ns
17 °C:1 nM vs. 20 °C:10 nM	0.9971	ns	0.0934	ns	0.4686	ns	0.999	ns	0.0014	**	0.5663	ns	0.0777	ns	0.9939	ns
17 °C:1 nM vs. 20 °C:100 nM	>0.9999	ns	0.1034	ns	0.942	ns	0.9849	ns	0.6043	ns	0.998	ns	0.0427	*	0.3251	ns
17 °C:10 nM vs. 17 °C:100 nM	0.1603	ns	0.3399	ns	>0.9999	ns	0.2712	ns	>0.9999	ns	>0.9999	ns	>0.9999	ns	0.9781	ns
17 °C:10 nM vs. 20 °C:0 nM	0.9994	ns	0.1804	ns	>0.9999	ns	0.8492	ns	0.0094	**	0.6427	ns	0.9989	ns	0.9767	ns
17 °C:10 nM vs. 20 °C:1 nM	0.9994	ns	0.213	ns	>0.9999	ns	0.9922	ns	0.005	**	0.948	ns	0.781	ns	>0.9999	ns
17 °C:10 nM vs. 20 °C:10 nM	0.9871	ns	0.2843	ns	0.4931	ns	>0.9999	ns	0.0009	***	0.0245	*	0.0777	ns	>0.9999	ns
17 °C:10 nM vs. 20 °C:100 nM	>0.9999	ns	0.309	ns	0.9522	ns	0.7795	ns	0.4776	ns	0.2435	ns	0.0427	*	0.8028	ns
17 °C:100 nM vs. 20 °C:0 nM	0.354	ns	0.9998	ns	>0.9999	ns	0.9504	ns	0.0076	**	0.5309	ns	0.9989	ns	>0.9999	ns
17 °C:100 nM vs. 20 °C:1 nM	0.0641	ns	>0.9999	ns	>0.9999	ns	0.0736	ns	0.004	**	0.8909	ns	0.781	ns	0.9864	ns
17 °C:100 nM vs. 20 °C:10 nM	0.0347	*	>0.9999	ns	0.4213	ns	0.3179	ns	0.0008	***	0.017	*	0.0777	ns	0.99	ns
17 °C:100 nM vs. 20 °C:100 nM	0.0939	ns	>0.9999	ns	0.9175	ns	0.9763	ns	0.4181	ns	0.1804	ns	0.0427	*	0.2959	ns
20 °C:0 nM vs. 20 °C:1 nM	0.9612	ns	>0.9999	ns	>0.9999	ns	0.4187	ns	>0.9999	ns	0.9965	ns	0.973	ns	0.9854	ns
20 °C:0 nM vs. 20 °C:10 nM	0.8608	ns	>0.9999	ns	0.4213	ns	0.8932	ns	0.9232	ns	0.4766	ns	0.2049	ns	0.9892	ns
20 °C:0 nM vs. 20 °C:100 nM	0.9887	ns	>0.9999	ns	0.9175	ns	>0.9999	ns	0.3626	ns	0.9924	ns	0.1195	ns	0.2912	ns
20 °C:1 nM vs. 20 °C:10 nM	>0.9999	ns	>0.9999	ns	0.518	ns	0.9835	ns	0.9852	ns	0.1804	ns	0.692	ns	>0.9999	ns
20 °C:1 nM vs. 20 °C:100 nM	>0.9999	ns	>0.9999	ns	0.9611	ns	0.3457	ns	0.2252	ns	0.8214	ns	0.5023	ns	0.7631	ns
20 °C:10 nM vs. 20 °C:100 nM	0.9992	ns	>0.9999	ns	0.9754	ns	0.8332	ns	0.0497	*	0.8909	ns	>0.9999	ns	0.7392	ns

Group C	Species name															
	S. acuminatus		*C. vulgaris*		*Pediastrum sp.*		*M. glauca*		*N. palea*		*N. vermicularis*		*C. cuspidata*		*S. minima*	
	P value		P value		P value		P value		P value		P value		P value		P value	
17 °C:0 nM vs. 17 °C:1 nM	0.4917	ns	>0.9999	ns	>0.9999	ns	0.9482	ns	>0.9999	ns	>0.9999	ns	>0.9999	ns	0.6556	ns
17 °C:0 nM vs. 17 °C:10 nM	0.822	ns	0.6669	ns	0.5908	ns	0.9744	ns	0.7134	ns	>0.9999	ns	>0.9999	ns	0.9998	ns
17 °C:0 nM vs. 17 °C:100 nM	0.0018	**	0.0015	**	0.9263	ns	0.901	ns	0.3444	ns	>0.9999	ns	0.9881	ns	0.0759	ns
17 °C:0 nM vs. 20 °C:0 nM	0.0004	***	0.001	**	0.8794	ns	0.9047	ns	0.3484	ns	>0.9999	ns	0.9999	ns	0.1462	ns
17 °C:0 nM vs. 20 °C:1 nM	0.0013	**	0.0006	***	0.6195	ns	0.9813	ns	0.8269	ns	0.9812	ns	0.5167	ns	0.4083	ns
17 °C:0 nM vs. 20 °C:10 nM	0.0006	***	0.0015	**	0.7315	ns	0.9988	ns	0.4987	ns	0.8981	ns	0.9987	ns	0.2829	ns
17 °C:0 nM vs. 20 °C:100 nM	0.0002	***	0.0008	***	0.0003	***	0.8043	ns	0.7181	ns	0.0064	**	0.8758	ns	0.2019	ns
17 °C:1 nM vs. 17 °C:10 nM	0.9986	ns	0.533	ns	0.6904	ns	>0.9999	ns	0.6408	ns	>0.9999	ns	>0.9999	ns	0.4063	ns
17 °C:1 nM vs. 17 °C:100 nM	0.0907	ns	0.001	***	0.9657	ns	0.3233	ns	0.407	ns	>0.9999	ns	0.9955	ns	0.806	ns
17 °C:1 nM vs. 20 °C:0 nM	0.018	*	0.0007	***	0.9353	ns	>0.9999	ns	0.4114	ns	>0.9999	ns	0.9992	ns	0.9454	ns

Group C	Species name															
	S. acuminatus		***C. vulgaris***		***Pediastrum sp.***		***M. glauca***		***N. palea***		***N. vermicularis***		***C. cuspidata***		***S. minima***	
	P value		P value		P value		P value		P value		P value		P value		P value	
17 °C:1 nM vs. 20 °C:1 nM	0.0629	ns	0.0004	***	0.7179	ns	>0.9999	ns	0.8808	ns	0.9812	ns	0.4441	ns	0.9998	ns
17 °C:1 nM vs. 20 °C:10 nM	0.0314	*	0.001	***	0.8196	ns	0.9993	ns	0.5717	ns	0.8981	ns	0.9955	ns	0.996	ns
17 °C:1 nM vs. 20 °C:100 nM	0.0116	*	0.0005	***	0.0004	***	>0.9999	ns	0.7858	ns	0.0064	**	0.8193	ns	0.9802	ns
17 °C:10 nM vs. 17 °C:100 nM	0.0306	*	0.0437	*	0.9964	ns	0.3913	ns	0.0191	*	>0.9999	ns	0.9947	ns	0.0337	*
17 °C:10 nM vs. 20 °C:0 nM	0.0058	**	0.0291	*	0.9991	ns	>0.9999	ns	0.0194	*	>0.9999	ns	0.9994	ns	0.0678	ns
17 °C:10 nM vs. 20 °C:1 nM	0.0208	*	0.0159	*	>0.9999	ns	>0.9999	ns	0.0981	ns	0.9812	ns	0.4559	ns	0.2185	ns
17 °C:10 nM vs. 20 °C:10 nM	0.0101	*	0.0437	*	>0.9999	ns	>0.9999	ns	0.0334	*	0.8981	ns	0.9963	ns	0.1415	ns
17 °C:10 nM vs. 20 °C:100 nM	0.0037	**	0.0229	*	0.0108	*	0.9993	ns	0.0674	ns	0.0064	**	0.8294	ns	0.0966	ns
17 °C:100 nM vs. 20 °C:0 nM	0.9859	ns	>0.9999	ns	>0.9999	ns	0.2592	ns	>0.9999	ns	>0.9999	ns	0.9073	ns	>0.9999	ns
17 °C:100 nM vs. 20 °C:1 nM	>0.9999	ns	0.9993	ns	0.9977	ns	0.4195	ns	0.9846	ns	0.9948	ns	0.1564	ns	0.9582	ns
17 °C:100 nM vs. 20 °C:10 nM	0.9988	ns	>0.9999	ns	0.9998	ns	0.6111	ns	>0.9999	ns	0.9508	ns	0.8393	ns	0.9915	ns
17 °C:100 nM vs. 20 °C:100 nM	0.951	ns	>0.9999	ns	0.0029	**	0.1784	ns	0.9969	ns	0.0091	**	0.4209	ns	0.9988	ns
20 °C:0 nM vs. 20 °C:1 nM	0.9971	ns	>0.9999	ns	0.9995	ns	>0.9999	ns	0.9854	ns	0.9991	ns	0.7533	ns	0.9965	ns
20 °C:0 nM vs. 20 °C:10 nM	>0.9999	ns	>0.9999	ns	>0.9999	ns	0.9965	ns	>0.9999	ns	0.9812	ns	>0.9999	ns	0.9999	ns
20 °C:0 nM vs. 20 °C:100 nM	>0.9999	ns	>0.9999	ns	0.0037	**	>0.9999	ns	0.9971	ns	0.013	*	0.9794	ns	>0.9999	ns
20 °C:1 nM vs. 20 °C:10 nM	>0.9999	ns	0.9993	ns	>0.9999	ns	>0.9999	ns	0.9987	ns	>0.9999	ns	0.8393	ns	>0.9999	ns
20 °C:1 nM vs. 20 °C:100 nM	0.9834	ns	>0.9999	ns	0.0099	**	0.9987	ns	>0.9999	ns	0.037	*	0.997	ns	0.9996	ns
20 °C:10 nM vs. 20 °C:100 nM	0.9994	ns	>0.9999	ns	0.0068	**	0.98	ns	>0.9999	ns	0.0728	ns	0.9937	ns	>0.9999	ns

Group C	Species name											
	S. vacuolatus		***C. polonicus***		***Phormidium sp.***		***P. galeata***		***B. braunii***		***C. accomoda***	
	P value		P value		P value		P value		P value		P value	
17 °C:0 nM vs. 17 °C:1 nM	0.1114	ns	0.9602	ns	>0.9999	ns	>0.9999	ns	0.9196	ns	0.6385	ns
17 °C:0 nM vs. 17 °C:10 nM	0.9988	ns	0.9993	ns	>0.9999	ns	>0.9999	ns	0.1032	ns	>0.9999	ns
17 °C:0 nM vs. 17 °C:100 nM	0.0003	***	<0.0001	****	0.9843	ns	>0.9999	ns	0.001	**	0.0223	*
17 °C:0 nM vs. 20 °C:0 nM	0.0001	***	0.0991	ns	0.1994	ns	>0.9999	ns	0.609	ns	0.0259	*
17 °C:0 nM vs. 20 °C:1 nM	0.0004	***	0.0116	*	0.0527	ns	>0.9999	ns	0.9565	ns	0.1003	ns
17 °C:0 nM vs. 20 °C:10 nM	0.0006	***	0.0026	**	0.0851	ns	0.654	ns	0.4123	ns	0.0584	ns
17 °C:0 nM vs. 20 °C:100 nM	<0.0001	****	<0.0001	****	<0.0001	****	0.8675	ns	0.0012	**	0.0345	*
17 °C:1 nM vs. 17 °C:10 nM	0.2842	ns	0.9994	ns	>0.9999	ns	>0.9999	ns	0.592	ns	0.7761	ns
17 °C:1 nM vs. 17 °C:100 nM	0.0806	ns	0.0005	***	0.9687	ns	>0.9999	ns	0.0001	***	0.4532	ns
17 °C:1 nM vs. 20 °C:0 nM	0.0351	*	0.4841	ns	0.1643	ns	>0.9999	ns	0.1086	ns	0.4965	ns
17 °C:1 nM vs. 20 °C:1 nM	0.1273	ns	0.0837	ns	0.0422	*	>0.9999	ns	0.3683	ns	0.8871	ns
17 °C:1 nM vs. 20 °C:10 nM	0.1675	ns	0.0201	*	0.0686	ns	0.7311	ns	0.0583	ns	0.7467	ns
17 °C:1 nM vs. 20 °C:100 nM	0.0054	**	<0.0001	****	<0.0001	****	0.9164	ns	0.0001	***	0.5843	ns
17 °C:10 nM vs. 17 °C:100 nM	0.0007	***	0.0002	***	0.9602	ns	>0.9999	ns	<0.0001	****	0.0356	*
17 °C:10 nM vs. 20 °C:0 nM	0.0003	***	0.2385	ns	0.1518	ns	>0.9999	ns	0.0031	**	0.0412	*
17 °C:10 nM vs. 20 °C:1 nM	0.0012	**	0.032	*	0.0386	*	>0.9999	ns	0.014	*	0.1528	ns
17 °C:10 nM vs. 20 °C:10 nM	0.0016	**	0.0073	**	0.0629	ns	0.5729	ns	0.0016	**	0.091	ns
17 °C:10 nM vs. 20 °C:100 nM	<0.0001	****	<0.0001	****	<0.0001	****	0.8051	ns	<0.0001	****	0.0546	ns

Group C	Species name											
	S. vacuolatus		*C. polonicus*		*Phormidium sp.*		*P. galeata*		*B. braunii*		*C. accomoda*	
	P value		P value		P value		P value		P value		P value	
17 °C:100 nM vs. 20 °C:0 nM	0.9998	ns	0.0265	*	0.6289	ns	>0.9999	ns	0.0359	*	>0.9999	ns
17 °C:100 nM vs. 20 °C:1 nM	>0.9999	ns	0.2044	ns	0.2403	ns	>0.9999	ns	0.008	**	0.9904	ns
17 °C:100 nM vs. 20 °C:10 nM	0.9998	ns	0.5679	ns	0.3516	ns	0.6241	ns	0.0683	ns	0.9995	ns
17 °C:100 nM vs. 20 °C:100 nM	0.8379	ns	0.7933	ns	0.0002	***	0.8458	ns	>0.9999	ns	>0.9999	ns
20 °C:0 nM vs. 20 °C:1 nM	0.9956	ns	0.9387	ns	0.9932	ns	>0.9999	ns	0.9923	ns	0.9947	ns
20 °C:0 nM vs. 20 °C:10 nM	0.9852	ns	0.5728	ns	0.9995	ns	0.6153	ns	>0.9999	ns	0.9998	ns
20 °C:0 nM vs. 20 °C:100 nM	0.9729	ns	0.0014	**	0.0049	**	0.8391	ns	0.0422	*	>0.9999	ns
20 °C:1 nM vs. 20 °C:10 nM	>0.9999	ns	0.9932	ns	>0.9999	ns	0.6974	ns	0.9435	ns	>0.9999	ns
20 °C:1 nM vs. 20 °C:100 nM	0.7028	ns	0.0129	*	0.0214	*	0.8963	ns	0.0095	**	0.9987	ns
20 °C:10 nM vs. 20 °C:100 nM	0.6077	ns	0.0551	ns	0.0129	*	0.9999	ns	0.0799	ns	>0.9999	ns

Supplementary References

1 Nübel, U., Garcia-Pichel, F. & Muyzer, G. PCR primers to amplify 16S rRNA genes from cyanobacteria. *Applied and Environmental Microbiology* **63**, 3327-3332, doi:doi:10.1128/aem.63.8.3327-3332.1997 (1997).

2 Hugerth, L. W. *et al.* Systematic Design of 18S rRNA Gene Primers for Determining Eukaryotic Diversity in Microbial Consortia. *PLoS ONE* **9**, e95567, doi:10.1371/journal.pone.0095567 (2014).

3 Vasselon, V., Rimet, F., Tapolczai, K. & Bouchez, A. Assessing ecological status with diatoms DNA metabarcoding: Scaling-up on a WFD monitoring network (Mayotte island, France). *Ecological Indicators* **82**, 1-12, doi:10.1016/j.ecolind.2017.06.024 (2017).

4 Godhe, A. *et al.* Quantification of Diatom and Dinoflagellate Biomasses in Coastal Marine Seawater Samples by Real-Time PCR. *Applied and Environmental Microbiology* **74**, 7174-7182, doi:doi:10.1128/AEM.01298-08 (2008).

5 Rimet, F. *et al.* Diat.barcode, an open-access curated barcode library for diatoms. *Scientific Reports* **9**, doi:10.1038/s41598-019-51500-6 (2019).

6 Vasselon, V. *et al.* Avoiding quantification bias in metabarcoding: Application of a cell biovolume correction factor in diatom molecular biomonitoring. *Methods in Ecology and Evolution* **9**, 1060-1069, doi:10.1111/2041-210x.12960 (2018).

7 Louca, S. *et al.* Function and functional redundancy in microbial systems. *Nature Ecology and Evolution* **2**, 936-943, doi:10.1038/s41559-018-0519-1 (2018).
